# Tropinone-Derived Alkaloids as Potent Anticancer Agents: Synthesis, Tyrosinase Inhibition, Mechanism of Action, DFT Calculation, and Molecular Docking Studies

**DOI:** 10.3390/ijms21239050

**Published:** 2020-11-28

**Authors:** Katarzyna Piechowska, Magdalena Mizerska-Kowalska, Barbara Zdzisińska, Joanna Cytarska, Angelika Baranowska-Łączkowska, Karol Jaroch, Kamil Łuczykowski, Wojciech Płaziński, Barbara Bojko, Stefan Kruszewski, Konrad Misiura, Krzysztof Z. Łączkowski

**Affiliations:** 1 Department of Chemical Technology and Pharmaceuticals, Faculty of Pharmacy, Collegium Medicum, Nicolaus Copernicus University, Jurasza 2, 85-089 Bydgoszcz, Poland; kpiechowska@cm.umk.pl (K.P.); cytar@cm.umk.pl (J.C.); kmisiura@cm.umk.pl (K.M.); 2 Department of Virology and Immunology, Maria Curie-Sklodowska University, Akademicka 19, 20-033 Lublin, Poland; magdalena.mizerska-dudka@poczta.umcs.lublin.pl (M.M.-K.); basiaz@poczta.umcs.lublin.pl (B.Z.); 3 Institute of Physics, Kazimierz Wielki University, ul. Powstańców Wielkopolskich 2, 85-090 Bydgoszcz, Poland; anxela@ukw.edu.pl; 4 Department of Pharmacodynamics and Molecular Pharmacology, Faculty of Pharmacy, Collegium Medicum, Nicolaus Copernicus University, Jurasza 2, 85-089 Bydgoszcz, Poland; karol.jaroch@cm.umk.pl (K.J.); k.luczykowski@cm.umk.pl (K.Ł.); bbojko@cm.umk.pl (B.B.); 5 Jerzy Haber Institute of Catalysis and Surface Chemistry, Polish Academy of Sciences, Niezapominajek 8, 30-239 Cracow, Poland; wojtek_plazinski@tlen.pl; 6 Medical Physics Division, Biophysics Department, Faculty of Pharmacy, Collegium Medicum, Nicolaus Copernicus University, Jagiellońska 13, 85-067 Bydgoszcz, Poland; skrusz@cm.umk.pl

**Keywords:** cancer, multiple myeloma, melanoma, tropinone, thiazole, mushroom tyrosinase, HOMO-LUMO, molecular docking

## Abstract

A new series of hybrid compounds with tropinone and thiazole rings in the structure was designed and synthesized as potential anticancer agents. They were tested against human multiple myeloma (RPMI 8226), lung carcinoma (A549), breast adenocarcinoma (MDA-MB-231), and mouse skin melanoma (B16-F10) cell lines. Toxicity was tested on human normal skin fibroblasts (HSF) and normal colon fibroblasts (CCD-18Co). The growth inhibition mechanism of the most active derivative was analyzed through investigation of its effect on the distribution of cell cycle phases and ability to induce apoptosis and necrosis in RPMI 8226 and A549 cancer cells. The tyrosinase inhibitory potential was assessed, followed by molecular docking studies. Compounds **3a**–**3h** show high anticancer activity against MDA-MB-231 and B16-F10 cell lines with IC_50_ values of 1.51–3.03 µM. Moreover, the cytotoxic activity of the investigated compounds against HSF and CCD-18Co cells was 8–70 times lower than against the cancer cells or no toxicity was shown in our tests, with derivative **3a** being particularly successful. The mechanism of action of compound **3a** in RPMI 8226 cell was shown to be through induction of cell death through apoptosis. The derivatives show ability to inhibit the tyrosinase activity with a mixed mechanism of inhibition. The final molecular docking results showed for IC_50_ distinct correlation with experiment.

## 1. Introduction

Cancer is a complex genetic and environmental disease and is currently the second largest cause of death after heart disease [1]. The number of cancer cases increases and is estimated to reach 19 million per year by 2024. Additionally, it is observed that the value of the cancer mortality rate is dangerously close to the incidence rate, which makes cancer a serious problem in a rapidly developing world. Currently, the most common types of cancer are lung, breast, colorectal, and prostate cancers. Lung and colorectal cancers are the two most common cancers in terms of incidence and mortality, both for males and females. As for males, the most common cause of death is prostate cancer, and for females it is breast cancer [1]. Another serious problem are the highly aggressive melanoma and multiple myeloma. In 2018, almost 300,000 cases of skin cancer were reported worldwide. The most common of cancer skin malignancies is melanoma, usually found on the skin exposed to sunlight, but also on the skin of the hands, feet, eye, and mucous membranes of the gastrointestinal tract. In melanoma cells, the process of melanin production catalyzed by tyrosinase is highly deregulated leading to its accumulation in cells. The TYR gene (encoding tyrosinase) is expressed only in specified cells that produce melanin. Thus, tyrosinase expression is limited only to melanocytic tumors such as most melanomas, desmoplastic melanoma (a relatively uncommon variant of melanoma), pigmented neurofibromas, or melanoma metastatic tumors to other organs [2]. Melanin belongs to a group of dyes that strongly absorb harmful UV radiation, which can protect melanoma cells during radiotherapy, and as the antioxidant may reduce the effectiveness of the chemotherapy used [3]. Melanin has also been found to have carcinogenic role in a pathway known as melanin chemiexcitation. As a result of activation of nitric oxide synthase (NOS) and NADPH oxidase by UV radiation, melanin is converted into a carbonyl form, which may form adducts with DNA or proteins, causing irreversible damage [4]. A possible solution to this problem is the use of tyrosinase inhibitors, inhibiting the sequential oxidation of L-tyrosine to L-DOPA-quinone—a substrate for melanin synthesis.

Multiple myeloma (MM) is a malignancy of plasma cells that leads to bone marrow failure, and consequently to anemia, decreased immunity leading to numerous infections, and kidney failure [5]. In recent years, the treatment of this type of cancer has improved significantly due to the introduction of new immunomodulating drugs from the IMIDs group, such as thalidomide, lenalidomide, or pomalidomide. Despite the use of new generation drugs, after the period of remission and relapses, most patients develop resistance to the drugs used, which in turn leads to their death [6,7]. Since the beginning of 2020 and the world spread of the SARS-CoV-2 virus, a new additional risk factor has appeared for patients with cancer diseases. Such patients are more likely to be infected with this virus, and, once infected, they experience a more severe course of the disease and have an increased risk of death. In particular, patients with MM, due to a weakened immune system, are more likely to be infected with SARS-CoV-2 [8].

Over the years of research, many methods of fighting cancer have been developed, such as chemotherapy, targeted therapy, monoclonal antibodies, and new immunotherapeutic drugs (personalized medicine). Promising results have been achieved with the emerging CAR-T Cell therapy (Chimeric-Antigen Receptor) and gene therapy that have recently been approved [9]. However, chemotherapy is still the most widely used tool for cancer treatment because it is readily available and inexpensive compared to modern treatments. Due to the fact that chemotherapeutic anticancer drugs often exhibit serious side effects, the development of effective drugs with high activity and minimal side effects on healthy cells is currently the main goal of medicinal chemists.

Tropinone (8-methyl-8-azabicyclo[3.2.1]octan-3-one), a typical component of the Solanaceae (*Cyphomandra betacea*) and Convolvulaceae families, containing the tropane core, is a key intermediate in the synthesis of many alkaloids such as hyoscyamine, scopolamine, atropine, and cocaine [10]. Although alkaloids containing a unique tropane core are used on a large scale as anticholinergic drugs in the treatment of neuromuscular disorders, Parkinson’s disease, and in poisoning with nerve agents, their antitumor activity has not been widely studied, especially when it comes to tropinone derivatives [11]. Currently, in the literature, we can find only three examples of research on tropinone derivatives as anticancer drugs, two of which concern benzylidene derivatives [12,13], and the third one reported by our research group and showing the importance of the thiazole ring in obtaining high antitumor activity [14].

Recent studies have shown that thiazole derivatives have a broad spectrum of biological and pharmacological activity, such as anticancer [15,16,17], antimicrobial [18], antituberculosis [19], anticonvulsant [20,21] activities, SARS-CoV **3c**L protease inhibition [22], and anti-*Toxoplasma gondii* activity [23,24]. Our research has shown that compounds including the thiazole system have excellent anticancer activity and additionally show low toxicity to healthy cells, which is a very important parameter in the design of new drugs. Bearing in mind our previous findings that the tropinone alkaloid is a very interesting scaffold for the design of anticancer drugs, we decided to modify the structure of our tropinone-thiazoles in order to achieve the highest possible activity against selected types of cancer, and that the compounds had a good toxicity profile.

The derivatives were selected to meet the Lipiński and Veber rules and additionally supported by quantum chemistry methods. Subsequently, the obtained compounds were tested against human multiple myeloma (RPMI 8226), human lung carcinoma (A549), human breast adenocarcinoma (MDA-MB-231), and mouse skin melanoma (B16-F10) cell lines, while the toxicity was tested on human normal skin fibroblasts (HSF) and human normal colon fibroblasts (CCD-18Co) using the 3-(4,5-dimethylthiazol-2-yl)-2,5-diphenyltetrazoliun bromide (MTT) assay.

To understand the growth inhibition mechanism of the most active derivative, we analyzed its effect on the distribution of cell cycle phases and examined the ability to induce apoptosis and necrosis in RPMI 8226 and A549 cancer cells. Finally, the tyrosinase inhibitory potential with an inhibitory mechanism was assessed, followed by molecular docking studies.

## 2. Results and Discussion

### 2.1. Chemical Synthesis and Chemoinformatics Data

**Scheme 1:**
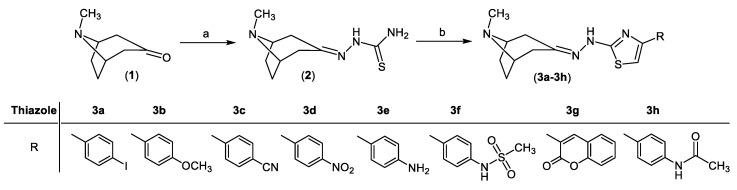
Synthesis of the target tropinone-thiazoles **3a**–**3h**. (**a**) thiosemicarbazide, glacial acetic acid, EtOH, reflux, 24 h (65%); (**b**) 4-substituted-bromoacetophenones, EtOH, reflux, 24 h (26–50%).

In this work, the corresponding tropinone-thiazole derivatives **3a**–**3h** were obtained starting from the key 2-(8-methyl-8-azabicyclo[3.2.1]acetate-3-ylidene)hydrazine-1-carbothioamide (**2**). An efficient synthesis of this compound has recently been developed in our research group [14]. Next, the target products **3a**–**3h** were synthesized by the reaction between carbothioamide **2** and the appropriate 4-substituted-bromoacetophenones in good yields (26–50%), and with high purity (Scheme 1). The structures of all the synthesized compounds were confirmed by several spectroscopic techniques, such as ^1^H NMR, ^1**3**^C NMR and LC-ESI-HRMS analysis. It is worth noting that the newly obtained tropinone analogues showed characteristic signals at (6.78–7.69) ppm derived from the hydrogen atom located in the thiazole ring. In the case of the ^13^C NMR spectra, we observe characteristic signals of about 109–111 ppm, and at about 170 ppm confirming the presence of the thiazole ring and the C=N group in the molecules, respectively.

To evaluate the drug-likeness of compounds, we used two of the most recognized filters, namely the Lipiński and Veber rules. These rules state that compounds have poor permeation and oral absorption if they have molecular weight (MW) > 500, lipophilicity values (logP) > 5, more than 5 hydrogen-bond donors (HBD), and more than 10 hydrogen bond acceptors (NHA). Veber’s rule introduces two additional parameters, that is the number of rotating bonds (NBR) and topological polar surface area (TPSA), the values of which cannot exceed 10 and 140 Å^2^, respectively [25]. The calculations performed with the SwissADME online tool [26] fully confirm that the tested compounds meet both, the Lipiński and Veber rules, which will positively affect their absorption and distribution through biological membranes, and that they can be orally active in humans (Table 1).

Quantum mechanical calculations are currently more and more widely employed in drug design to support experimental search for new drugs. Quantum chemistry methods, in particular those based on the density functional theory (DFT), are helpful for accurate description of intermolecular interactions and evaluation of the potential drug’s reactivity [27]. Within the present work, the DFT approximation is employed in the calculation of the highest occupied molecular orbital (HOMO) and the lowest unoccupied molecular orbital (LUMO) shape and energy, as well as the molecular electrostatic potential (MEP) surfaces. HOMO and LUMO are the two main orbitals involved in chemical reactions. Based on the values of their energies, denoted as *E_HOMO_* and *E_LUMO_*, respectively, the HOMO-LUMO energy gap Δ*E*, ionization potential *IP*, electron affinity EA, hardness *η*, softness *S*, and electronegativity *χ* can be found. The Δ*E* defined as:

Δ*E* = *E_LUMO_* − *E_HOMO_*

gives insight into the molecule’s chemical stability. Its size yields information on excitation energy of investigated species, helping in understanding molecule’s reactivity. The *IP* of given system is the energy needed to remove an electron from a molecule, and is related to the HOMO energy as follows:

*IP* = −*E_HOMO_*

The lower *IP* corresponds to an easier electron removal, and thus larger tendency to donate electrons. Instead, molecule’s *EA* is the energy released upon gaining an electron by a molecule, and can be obtained from the LUMO energy:

*EA* = −*E_LUMO_*

Its value informs about molecule’s reductant properties. Molecule’s electronegativity χ represents its ability to attract electrons [28]:

*χ* = (*IP* + *EA*)/2

Molecule’s hardness *η* is defined as [28]:

*η* = (*IP* − *EA*)/2 = 1/*S*

Large values of *η* (and thus low values of softness S) correspond to large resistance to change in the number of electrons. Estimation of molecular reactivity and prediction of molecular sites susceptible to nucleophilic and electrophilic attacks can be assisted by evaluation of the molecular electrostatic potential (MEP) surfaces. Let us assume a positive test charge at a given point. The MEP is a force acting on this test charge due to the molecule’s electron cloud. In practice, MEP surfaces are formed through mapping the molecular electrostatic potential onto some surface reflecting molecule boundaries, e.g., electron density isosurface. The electron-rich and electron-deficient regions of MEP surface are presented using different colors, enabling recognition of regions susceptible to nucleophilic and electrophilic attacks.

**Figure 1:**
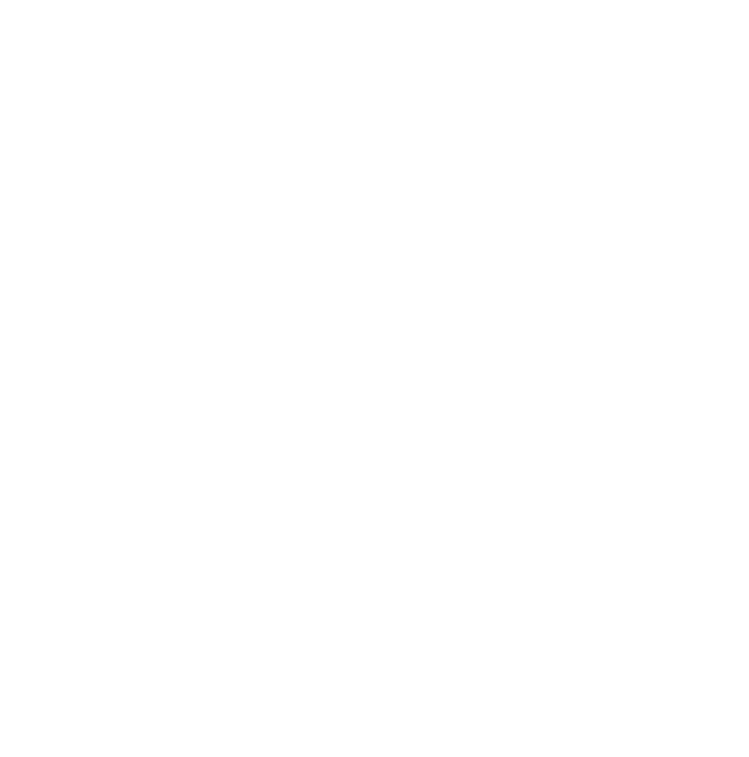
Molecular orbital distribution plots of HOMO-LUMO for compound **3h** at the B3LYP/6-311G** level.

The results of quantum chemistry calculations are presented in Table 1. The shape of HOMO and LUMO orbitals for one of the most active compounds, that is compound **3h**, are presented in Figure 1, and for all eight investigated molecules in the Supplementary Materials. The HOMO-LUMO energy gap values are relatively small, between 3.35 and 4.31 eV. Automatically, the corresponding hardness values are low (between 1.67 and 2.15 eV), suggesting high reactivity of the investigated drugs. In general, the compounds showing the larger activity are those with the larger absolute values of HOMO-LUMO energy gap and relatively large hardness, while those less active have lower values of Δ*E* and *η*. Compounds that do not follow this trend are one of the less active **3b** and one of the most active **3d**, corresponding to the largest and the smallest HOMO-LUMO energy gap, respectively. It should be however stressed that Δ*E* and *η* parameters correlate with molecule’s chemical reactivity in general, but are not necessarily reflected in its biological activity, as the latter depends also on many other parameters and effects.

The largest values of *IP*, *EA*, and χ were obtained for compound **3d**, while the smallest was for compound **3e**. The difference between *IP*, *EA*, and χ values for these two systems are 0.87, 1.72, and 1.30 eV, respectively. We can thus conclude that electron removal would be the easiest for molecule **3e**, which is also the most active (has the lowest IC_50_ value) among the investigated compounds. Molecule **3d**, with its low energy HOMO, the smallest energy gap, and the largest softness, accepts electrons more readily than other systems under study, which most likely will be reflected by its reactivity. The plots of LUMO orbitals (see Supplementary Materials) reveal that the lower energy LUMOs are located mainly on phenylthiazole moiety, and with the increase of energy they shift towards hydrazone moiety, covering almost the whole molecule.

**Figure 2:**
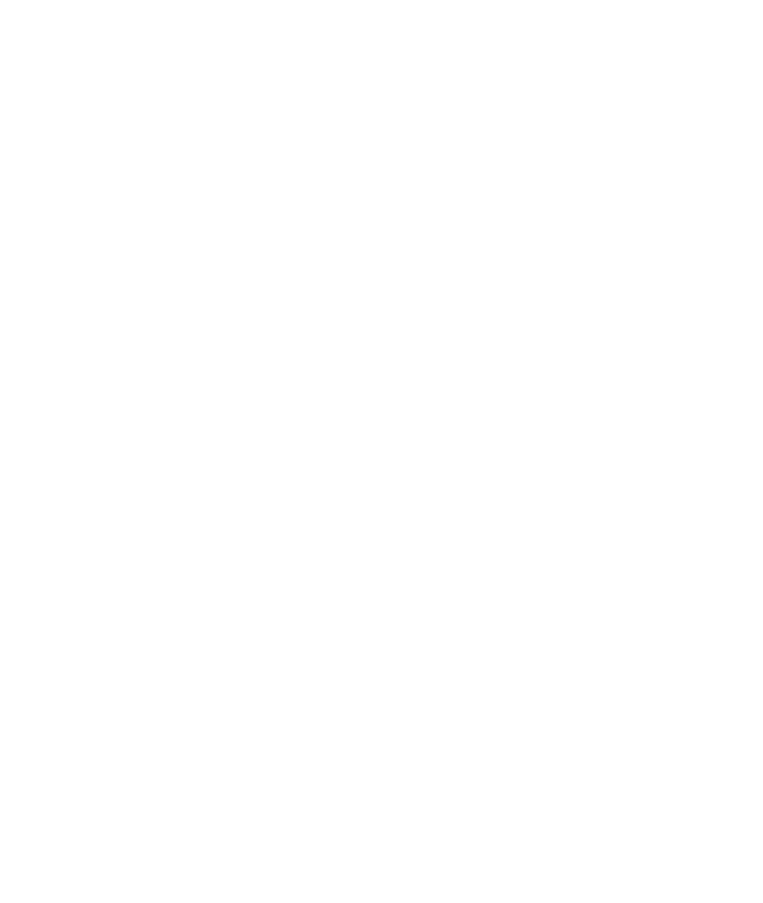
Molecular electrostatic potential (MEP) surface for compounds **3a**–**3h**.

Analysis of the MEP surfaces show that the electron-rich regions are located around oxygen atoms present in substituents, and around CN group in **3c** molecule. Additional electron-rich region around thiazole moiety is observed for molecules **3b**, **3e**, and **3h**. Electron-deficient regions are located around hydrazone moiety, and additionally around –NH_2_ in molecule **3e**, as well as –NH– substituent in **3f** and **3h** (Figure 2).

The molecular properties collected in Table 1 as well as the experimentally-inferred IC_50_ values (expressed as pIC_50_ = −log(IC_50_)) were subjected to further analysis in order to test their mutual correlation and possible contribution in the prospective quantitative structure–activity relationship (QSAR) model. Table S1 contains the Pearson’s correlation matrix calculated for 14 molecular descriptors and pIC_50_. The obtained values of correlation coefficients (R) suggest that the degree of intercorrelation between descriptors is very broad and dependent on the considered descriptors’ pair. In addition, the correlation between pIC_50_ and descriptors varies from very weak (TPSA) to moderate (HBD). Clearly, no single descriptor is capable to provide the quantitatively accurate relationship against pIC_50_. The minimal, 3-parameter QSAR models were constructed by using the preselected set of descriptors, exhibiting the highest correlation with pIC_50_ (|R| > 0.4). The additional preselection condition was relatively weak correlation between any possible pair of descriptors (|R| > 0.6). Accepting such conditions resulted in only several possible triplets of descriptors; among them, the two displayed similarly high performance in constructing QSAR models. The models relied on multiple linear regression in order to find the coefficients in the assumed linear dependence of pIC_50_ on the tested descriptors. Those two models can be described by the following relationships:

pIC_50_ = −0.1306 *logP* − 0.06104 *E_HOMO_* − 0.08138 *HBA* + 4.2333

**Figure 3:**
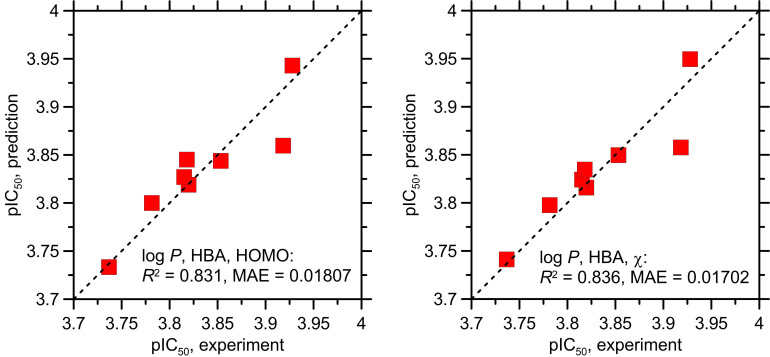
The comparison of the experimental pIC_50_ = −log(IC_50_) values with the predictions of the two 3-parameter QSAR models. See details in the text.

pIC_50_ = −0.1299 *logP* + 0.04608 *χ* − 0.08499 *HBA* + 4.4194

and are characterized by nearly the same values of mean absolute error of prediction (MAE equal to 0.01807 and 0.01702, respectively) and determination coefficients (R^2^ equal to 0.831 and 0.836, respectively). Figure 3 shows the comparison of the experimental pIC_50_ values with the associated predictions offered by the two above QSAR models. Both models indicate the significant contributions of both the magnitude of molecule lipophilicity and the number of hydrogen bond acceptors in its structure. The remaining pair of descriptors (*E_HOMO_* and *χ*) is mutually convoluted through the *E_LUMO_* value and strongly correlated (R = −0.987). Thus, their similar role as complementing parameter in the QSAR models is reasonable.

### 2.2. Effect of Compounds ***3a**–**3h*** on Cancer Cell Proliferation and Viability of Normal Cells

The synthesized analogues **3a**–**3h** were screened for their antiproliferative activity against four different types of cancer cell lines (i.e., human multiple myeloma (RPMI 8226), human lung carcinoma (A549), human breast adenocarcinoma (MDA-MB-231), and mouse skin melanoma (B16-F10) within 96 h or 72 h via MTT assay. In turn, to predict the acute cytotoxicity of these compounds towards normal cells, we examined their influence on the viability of human normal skin fibroblasts (HSF) and human normal colon fibroblasts (CCD-18Co) in confluent monolayers after 24 h of incubation. The obtained results showed that tropinone–thiazole hybrids **3a**–**3h** have high activity against MDA-MB-231 cells with IC_50_ values between 2.26–2.90 µM, and also high activity against B16-F10 cells with IC_50_ values between 1.51–3.03 µM (Table 2). In both cases, this activity is comparable to or even higher than the activity of the reference anticancer agent, chlorambucil, as well as two orders of magnitude higher activity than the standard tyrosinase inhibitor ascorbic acid, which has anticancer activity against B16F10 cell line with LD_50_ of 800 µM [29]. Moreover, compounds **3a**–**3h** either showed the cytotoxic activity against the normal cells from 8 to 70 times lower than against the cancer cells or showed no toxicity in tests (higher CC_50_ values for both normal cell lines). Additionally, among the compounds tested, compound **3a** showed the greatest antiproliferative activity against RPMI 8226 and A549 cell lines (Table 2). The IC_50_ values of compound **3a** for the RPMI 8226 and A549 cells at 96 h were 9.32 μM and 13.81 μM, respectively. The CC_50_ values of compound **3a** for the HSF and CCD-18Co cells were higher and amounted to 19.23 µM and 27.76 µM, respectively (Table 2), suggesting more selective activity of this compound against cancer cells. Similarly, compound **3d** and **3g** showed good antiproliferative activity against the RPMI 8226 cells with IC_50_ of 17.87 µM and 23.16 µM, respectively. However, these compounds exhibited weaker activity against the A549 cells with IC_50_ of 30.99 µM and 29.29 µM, respectively. Moreover, the cytotoxic activity of compounds **3d** and **3g** against the normal cells was lower than against the cancer cells (higher CC_50_ values for both normal cell lines). Compounds **3b** and **3c** showed moderate antiproliferative activity against the RPMI 8226 cells (IC_50_ values 42.19 µM and 31.70 µM, respectively), but weakly inhibited the proliferation of the A549 cells (IC_50_ values 132.7 µM and 74.93 µM, respectively). Compounds **3e**, **3f**, and **3h** had very low antiproliferative activity against the cancer cells (Table 2) and showed weak (compound **3f** against the HSF cells) or no toxicity (compounds **3e** and **3h** against the HSF cells and compounds **3e**, **3f**, and **3h** against CCD-18Co cells) against the normal cells (Table 2).

In the present study, tropinone–thiazole hybrids **3a**–**3h** exerted antiproliferative activity against the tested cancer cell lines, although different sensitivity depending on the cell line type was observed. This phenomenon, also observed in our previous study [14], strongly suggest a cell-type specific effect of these compounds, probably related to distinct genotypic and phenotypic characteristic of the tested cancer cell lines.

The cell cycle, the process by which cells progress and divide, is tightly regulated in normal cells through growth-regulatory signals and several cell cycle-associated protein regulators. Loss of normal cell cycle control results in uncontrolled cell proliferation that is one of the hallmarks of cancer [30]. Cell cycle perturbation plays a critical role in tumorigenesis; therefore, cell cycle inhibitors could be potential therapeutic drugs for the treatment of cancers [31]. Another hallmark of cancer is evasion of apoptosis, which is consequent dysregulation of mechanisms of this physiological mode of programmed cell death [30]. The induction of apoptosis is a good approach for identification of the anticancer potential of particular compounds.

**Figure 4:**
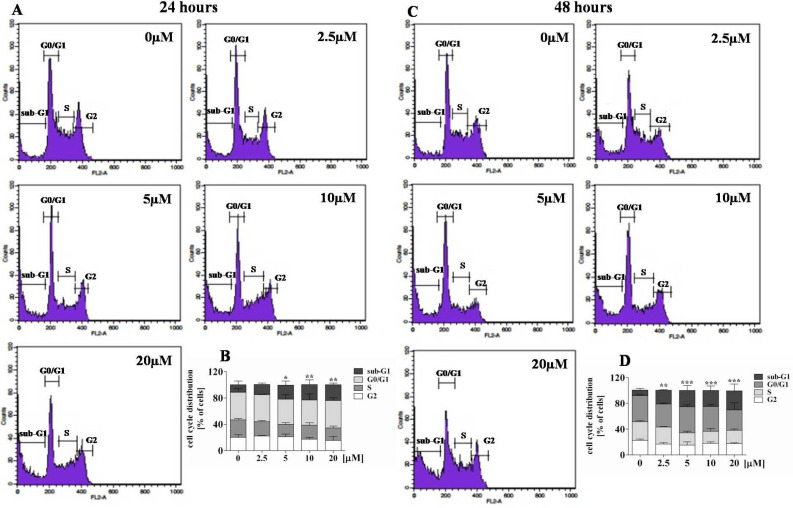
Effect of compound **3a** on cell cycle distribution in the RPMI 8226 cell line. After the treatment with various concentrations of compound **3a** for 24 and 48 h, the cells were stained with propidium iodide and analyzed by flow cytometry. Representative DNA histograms after 24-h (**A**) and 48-h (**C**) treatment. Statistical analysis of the percentages of cells in the sub-G1, G0/G1, S, and G2 phases after 24-h (**B**) and 48-h (**D**) treatment. Data (*n* = 6 for each concentration) are presented as the mean ± SD of three independent experiments; * *p* < 0.05, ** *p* < 0.01 and *** *p* < 0.001 in comparison to the control; one-way ANOVA test.

**Figure 5:**
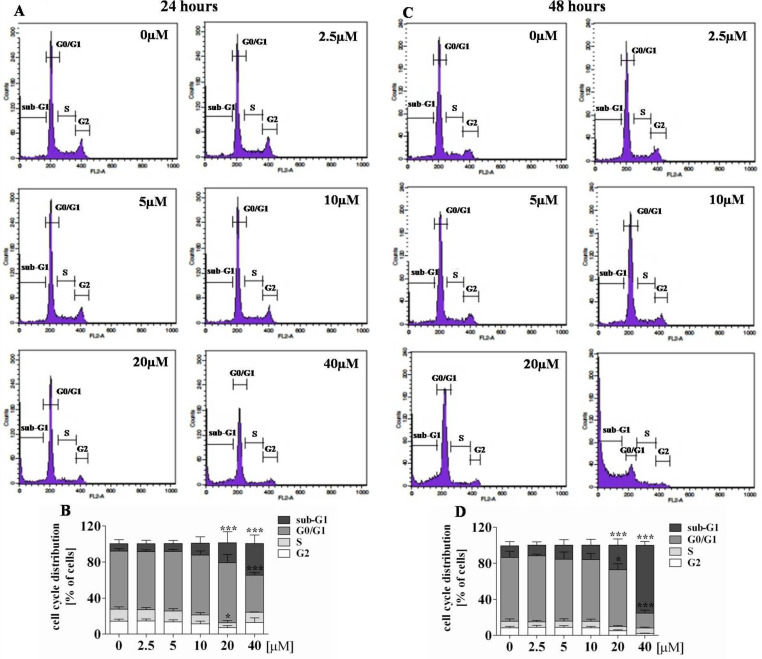
Effect of compound **3a** on cell cycle distribution in the A549 cell line. After the treatment with various concentrations of compound **3a** for 24 and 48 h, the cells were stained with propidium iodide and analyzed by flow cytometry. Representative DNA histograms after 24-h (**A**) and 48-h (**C**) treatment. Statistical analysis of the percentages of cells in the sub-G1, G0/G1, S, and G2 phases after 24-h (**B**) and 48-h (**D**) treatment. Data (*n* = 6 for each concentration) are presented as the mean ± SD of three independent experiments; * *p* < 0.05, ** *p* < 0.01 and *** *p* < 0.001 in comparison to the control; one-way ANOVA test.

To gain insight into the mechanism of the growth inhibitory potential of compound **3a** against the RPMI 8226 and A549 cells, we analyzed its influence on the distribution of cell-cycle phases in both cell line cultures after 24 and 48 h. As can be seen in Figure 4 and Figure 5, the exposure of the RPMI 8226 (Figure 4A–D) and A549 (Figure 5A–D) cells to compound **3a** did not result in arrest and did not increase the number of cells in G0/G1, S, or G2 phases of the cell cycle. In contrast, it induced a concentration- and time-dependent increase in the cellular content in the sub-G1 phase (Figure 4B,D and Figure 5B,D, Tables S2 and S3), which corresponds to apoptotic cells with fractional DNA content. Thus, the results indicate that compound **3a** can induce apoptosis in cancer cells, but not via the cell cycle arrest.

**Figure 6:**
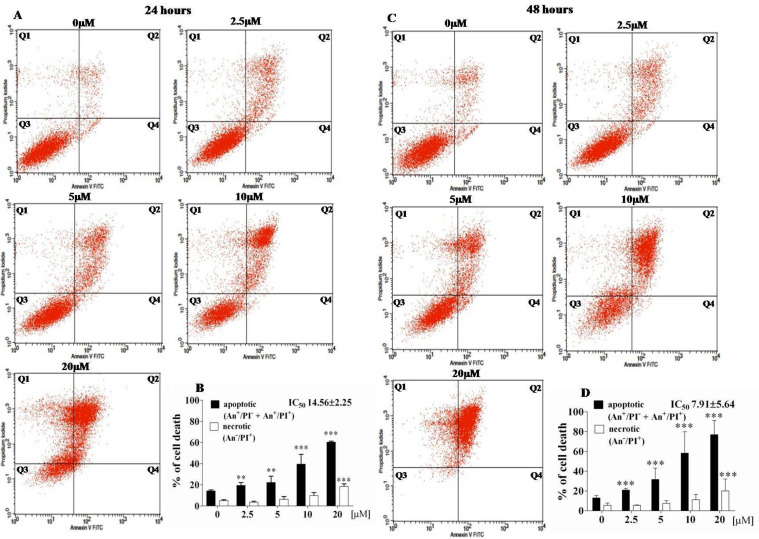
Effect of compound **3a** on apoptosis/necrosis induction in the RPMI 8226 cells. After the 24-h (**A**) and 48-h (**C**) exposure to the different concentrations of compound **3a**, the cells were stained with Annexin (An) V-FITC/propidium iodide (PI) and examined with flow cytometry. Quadrant Q1 represents An^−^/PI^+^ necrotic cells, Q2—An^+^/PI^+^ late apoptotic cells, Q3—An^−^/PI^−^ viable cells, Q4—An^+^/PI^−^ early apoptotic cells. Histogram representation of the quantitative percentage of total apoptotic cells (early + late apoptosis) and necrotic cells in the control and compound **3a**-treated cell cultures after the 24-h (**B**) and 48-h (**D**) treatment. All experiments were repeated independently at least three times, and data (*n* = 6 for each concentration) are expressed as mean ± SD; ** *p* < 0.01 and *** *p* < 0.001 in comparison to the control; one-way ANOVA test.

**Figure 7:**
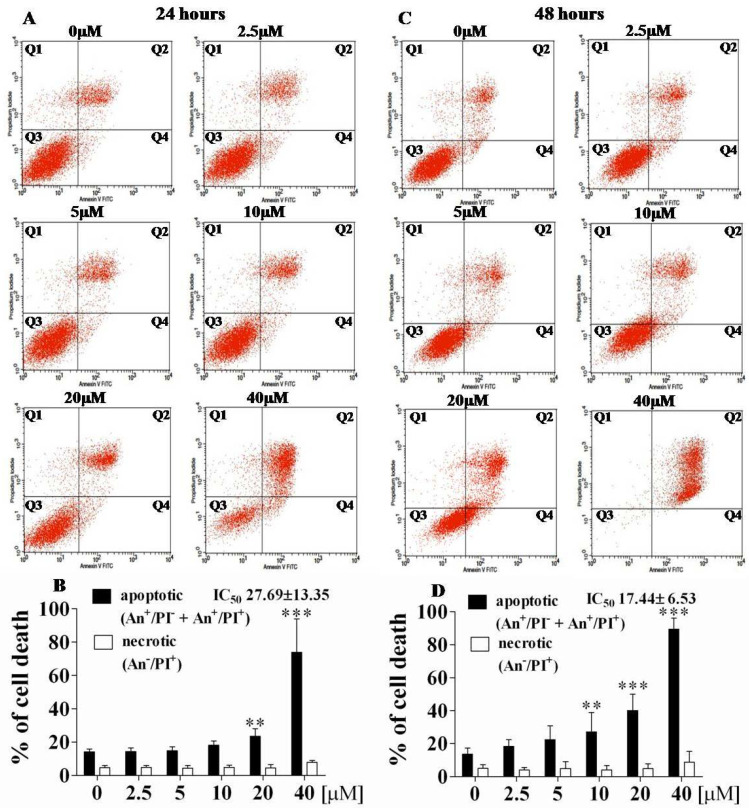
Effect of compound **3a** on apoptosis/necrosis induction in the A549 cells. After the 24-h (**A**) and 48-h (**C**) exposure to the different concentrations of compound **3a**, the cells were stained with Annexin (An) V-FITC/propidium iodide (PI) and examined with flow cytometry. Quadrant Q1 represents An^−^/PI^+^ necrotic cells, Q2—An^+^/PI^+^ late apoptotic cells, Q3—An^−^/PI^−^ viable cells, Q4—An^+^/PI^−^ early apoptotic cells. Histogram representation of the quantitative percentage of total apoptotic cells (early + late apoptosis) and necrotic cells in the control and compound **3a**-treated cell cultures after the 24-h (**B**) and 48-h (**D**) treatment. All experiments were repeated independently at least three times, and data (*n* = 6 for each concentration) are expressed as mean ± SD; ** *p* < 0.01 and *** *p* < 0.001 in comparison to the control; one-way ANOVA test.

To verify whether compound **3a** can trigger apoptosis and necrosis in cancer cells, the RPMI 8226 and A549 cell lines were treated with different concentrations of compound **3a** for 24 and 48 h. As revealed by Annexin V-FITC/PI double staining, compound **3a** was found to trigger RPMI 8226 and A549 cell death mainly via the apoptotic process. It induced apoptosis in both cell lines in a concentration- and time-dependent manner. The IC_50_ values of compound **3a** for apoptosis in the RPMI 8226 cells (defined as concentrations of the compound necessary to induce apoptosis in 50% of cells) at 24 and 48 h were 14.56 µM and 7.91 µM, respectively. Significant induction of apoptosis in the RPMI 8226 cells was observed even at a dose of 2.5 μM of compound **3a**. In contrast, necrosis was only slightly increased when the RPMI 8226 cells were incubated with 20 μM of compound **3a** (Figure 6A–D and Table S4). However, compound **3a** was a weaker inducer of apoptosis in the A549 cells, as the IC_50_ values for apoptosis in this cell line at 24 and 48 h were 27.69 µM and 17.44 µM, respectively. Moreover, compound **3a** did not induce necrosis in the A549 cells even at the highest concentration, i.e., 40 µM (Figure 7A–D and Table S5).

### 2.3. Tyrosinase Inhibitory Activity

Subsequently, all the derivatives obtained were explored for their tyrosinase inhibitory ability using L-DOPA as a substrate. The obtained results were compared with the best known tyrosinase inhibitor, which is ascorbic acid. Interestingly, compound **3e** containing the amino group as well as compound **3h** containing the acetylated amino group showed the best tyrosinase inhibitory activity, with IC_50_ equal to 118.06 and 120.71 µM, respectively (Table 3). These compounds are also three times more active than standard ascorbic acid (IC_50_ 386.5 µM). The compound **3d** also exhibited good activity with IC_50_ equal to 140.21 µM compared to standard ascorbic acid. Our research also indicated that compound **3g** containing methanesulfonamide substituent showed the smallest tyrosinase inhibition effect, with IC_50_ equal to 180.34 µM.

**Figure 8:**
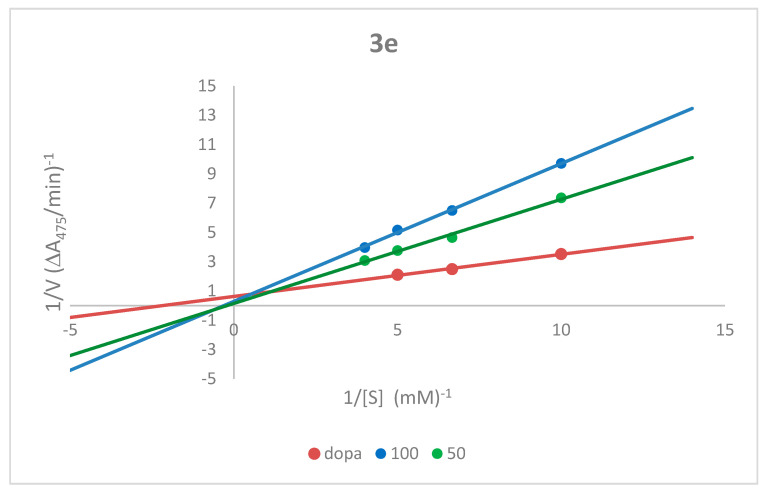
Lineweaver-Burk plots for tyrosinase inhibition for compounds **3e** at concentration 200 µM using L-DOPA as substrate at concentrations 100, 150, 200 and 250 µM.

To understand the mechanism of mushroom tyrosinase inhibition by new compounds **3a**–**3h**, enzyme inhibition kinetic studies were performed. The mechanism of tyrosinase inhibition was determined using Lineweaver–Burk plot, which is a useful graphical method for analysis of the Michaelis–Menten equations used to determine the inhibition constant K_M_. This graphical representation showed that compounds **3a**–**3h** exhibited a mixed mechanism of tyrosinase inhibition, meaning that investigated molecules are able to bind to the enzyme even if it already bound other substrate (Figure 8).

### 2.4. Docking Study

**Figure 9:**
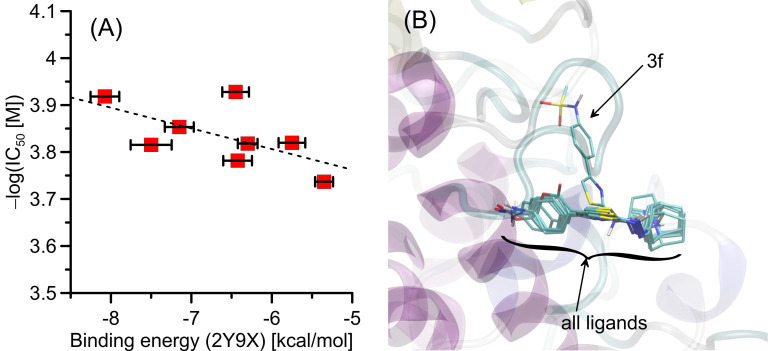
(**A**) The correlation between the binding energies calculated for 2y9x interacting with a set of ligand molecules (**3a**–**3h** and ascorbate ion) with the −log(IC_50_) values. The numerical values can be found in Table 4. (**B**) The superposition of poses of all ligands found during docking study. Compound **3f** may exhibit the two alternative poses, only slightly differing by binding energies (see discussion in the text).

Binding energies found during docking simulations are given in Table 4 and graphically illustrated in Figure 9. The similar magnitude of the binding energies obtained for compounds **3a**–**3h** can be noted (−5.4–−8.1 kcal/mol). Much weaker binding strength was found for ascorbic acid (considered as the three distinct tautomeric forms of ascorbate ion), i.e., −4.4 kcal/mol. Whereas the order of interaction strengths is correctly reproduced for the compounds exhibiting higher values of IC_50_, the docking results are at odds with the experiment when considering the identification of the compound exhibiting the lowest IC_50_ value and highest potency (namely: compound **3e**). Instead of that, the second most potent compound **3h** is identified as that displaying the highest binding energy. This may partially originate from rather minor differences between the experimental IC_50_ values; when neglecting ascorbic acid, they vary within the limits of 118–183 µM, while the difference between the two most potent compounds is <3 µM. Nevertheless, the binding energies calculated for all set of compounds show distinct correlation with −log(IC_50_) calculated from the experimentally determined values of IC_50_ (see Figure 9). Thus, we concluded that an agreement between the theoretical and experimental results is sufficient to perform some more detailed analysis, focused on identifying the pattern of ligand–protein interactions.

**Figure 10:**
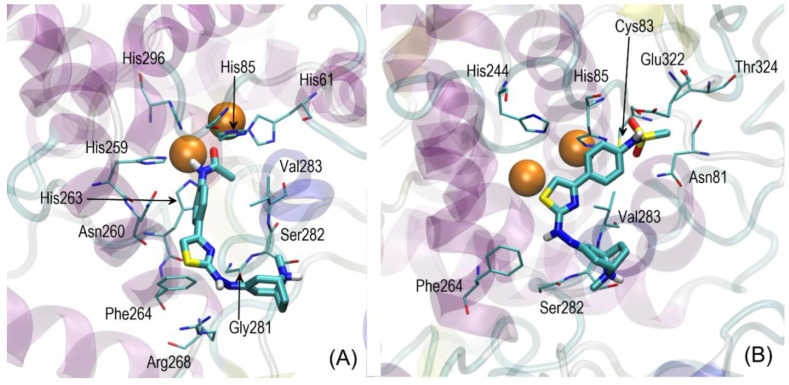
(**A**) The energetically-favorable location of the ligand molecule **3h** bound to the 2y9x structure. (**B**) The alternative position of binding found only for the **3f** ligand. The ligand molecules are shown as thick sticks whereas all the closest amino-acid residues are represented by thin sticks. Orange balls represent the copper ions, present in the protein crystal structure. The description of the interaction types is given in the text.

The results of the docking studies have also been analyzed with respect to the mechanistic interaction patterns that may be significant in the context of interpretation of the obtained binding energy values. The summary given below relies on analyzing the ligand–protein contacts that occur if the distance between any corresponding atom pair is smaller than the arbitrarily accepted value of 0.38 nm. We have found that nearly all the studied ligands dock to the protein structure in a very similar manner (see Figure 10). The only exception is compound **3f**, which exhibits the two alternative orientations, significantly differing in both the molecular conformation of the ligand itself and the ligand–protein set of contacts; one of those orientations is analogous to those characteristic of all remaining ligands. However, it is worth mentioning that the binding energies associated with those two poses are extremely similar and, thus, the discrimination of one of the orientations over another does not influence the numerical values collected in Table 4 and discussed above. Nevertheless, due to lack of evidence that any of those two possibilities should be neglected, we discuss them both. Thus, the description provided below concerns the two separate cases: (i) the compound **3h** exhibiting the highest binding energy for which the found interactions pattern is representative for all studied compounds, within the accuracy limits resulting from the topological divergences within ligand structure; (ii) compound **3f** with the unique interaction pattern. The graphical illustration of the docking results is given in Figure 10.

Considering the contacts common for all studied ligands, it is worth noting that the interactions involving the –R substituent (variable from one ligand to another) involve the copper ions, present in the crystal structure of protein. This is in contrast with the results obtained in our previous study for the same protein where the ligand-Cu distance was large enough to neglect the possibility of some specific interactions involving Cu [23]. Here, it cannot be excluded and such interactions (e.g., coordinate bonds or charge transfer) can be treated by docking methodology only in an approximate manner. This may be another reason for a limited correlation between calculated binding energies and experimental data. In general, each of the –R substituents contain the electronegative part (either single atom or group of different atoms); thus, some part of related attractive interactions can be ascribed to a purely electrostatic component. In spite of the presence of several His residues in the vicinity of the –R moiety (e.g., His61, His85, His296, His259, His263), their spatial arrangements with respect to the ligand suggest that the observed contacts are just an opportunistic consequence of close proximity of the –R moiety and Cu ions, and do not result from any specific –R-His interactions. Only in the case of His85 and His259 and some of the ligands, additional hydrogen bonding-mediated contacts may occur, where –R plays a role of an acceptor. However, this additional contribution is not clearly reflected in the associated binding energies. Apart from those contacts, non-polar parts of the –R group can, in some cases, exhibit interactions with Val283; they can either be of the H-π type (and involving the phenyl part of the –R group) or just non-specific hydrophobic contacts (involving the methyl part of –R). Furthermore, the phenyl ring of the –R moiety interacts with Asn260 (possibly by the lone pair-π attraction). The thiazole ring of the ligand maintains close contact with Phe264 by the attractive H-π stacking and the neighboring hydrazone group creates hydrogen bonding with Arg268. Finally, the amine group located at the bulk, aliphatic moiety of the ligand, forms hydrogen bond with Ser282. In spite of relatively close proximity of Gly281 and the aliphatic group of the ligand, no specific interactions were observed within this pair.

The individual interaction pattern exhibited by compound **3f** is characterized by a larger distance between Cu ions and the −NHSO_2_Me group in comparison to previously discussed interactions involving –R substituent. Instead, the −NHSO_2_Me moiety interacts with Thr324, Glu322, and Asn81. The two latter contacts are maintained through the presence of hydrogen bonding whereas the former one speaks for the occurrence of weaker, hydrophobic interactions between methyl groups. Cys83, located close to −NHSO_2_Me does not seem to exhibit any specific types of interactions with ligand. At the same time, phenyl ring of the ligand interacts with His85 and His244 by the H-π stacking. Interestingly, the thiazole ring does not exhibit any close contacts with protein. The hydrazine moiety is located close to Phe264, which suggests the existence of the H-π interactions. Finally, the location of aliphatic moiety is stabilized by the contacts with hydrophobic sidechains of Val283 while its amine substituent creates hydrogen bonding with neighboring Ser282. Note that this binding pattern is closer to that observed in our previous study and described in [23] in comparison to the poses discussed above and corresponding to the remaining ligands.

The first described interaction pattern is common for all studied ligands that reflect the similar values of the calculated binding energy (all values differ by no more than 2.7 kcal/mol). The structural differences between ligands are limited to the substitution of the phenyl moiety inherent to the –R group and, in one case, in alternative type of the aromatic part of –R. Thus, the divergences between binding energies observed within the group of compounds **3a**–**3h** can be ascribed to the interactions between the –R substituent and the Cu ions, with possible contributions from His85, His259, Asn260, and Val282.

## 3. Materials and Methods

### 3.1. Chemistry

All experiments were carried out under air atmosphere. Reagents were generally the best quality commercial-grade products and were used without further purification. ^1^H NMR (700 MHz) and ^13^C NMR (100 MHz) spectra were recorded on a Bruker Avance III multinuclear instrument. LC-ESI-HRMS was performed by the Laboratory for Analysis of Organic Compounds and Polymers of the Centre for Molecular and Macromolecular Studies of the Polish Academy of Science in Łódź. Melting points were determined in open glass capillaries and are uncorrected. Analytical TLC was performed using Macherey–Nagel Polygram Sil G/UV_254_ 0.2 mm plates. Tropinone, thiosemicarbazide, and appropriate bromoketones were commercial materials (Aldrich).

#### 3.1.1. Synthesis and Structural Characterization

##### 2-(8-Methyl-8-azabicyclo[3.2.1]octan-3-ylidene)hydrazine-1-carbothioamide (**2**)

The compound 2 was obtained according to the procedure described previously [14].

General experimental procedure for synthesis of tropinone-thiazole derivatives **3a**–**3h**:

Carbothioamide 2 (0.212 g, 1.0 mmol) was added to a stirred solution of 2-bromo-1-(4-iodophenyl)-ethanone (0.325 g, 1.0 mmol) in absolute ethyl alcohol (20 mL). The reaction mixture was stirred under reflux for 20 h. Next, the reaction mixture was added to water (50 mL) and neutralized with NaHCO_3_ solution. The product was extracted with dichloromethane (2 × 100 mL), the solvent was evaporated in vacuo, and the product was purified on silica gel column chromatography (230–400 mesh) using (dichloromethane/methanol, 80:20) to afford the desired product **3a** in 48% (0.31 g) isolated yield. The similar procedure was adopted for the synthesis of other compounds **3b**–**3h**.

##### 4-(4-Iodophenyl)-2-(2-(8-methyl-8-azabicyclo[3.2.1]octan-3-ylidene)hydrazinyl)thiazole (**3a**)

Yields 48%; mp 92–93 °C. ^1^H NMR (700 MHz, DMSO-d_6_), δ (ppm): 1.29–1.35 (m, 1H, CH_2_); 1.42–1.49 (m, 1H, CH_2_); 1.88–1.97 (m, 2H, 2CH_2_); 2.08 (d, 1H, CH_2_, J = 14.7 Hz); 2.23 (dd, 1H, CH_2_, J = 3.5 Hz, J = 16.1 Hz); 2.28 (s, **3h**, CH_3_); 2.59 (dd, 1H, CH_2_, J = 3.5 Hz, J = 16.0 Hz); 2.66 (d, 1H, CH_2_, J = 15.4 Hz); 3.24 (s, 2H, 2CH); 7.29 (s, 1H, CH); 7.63 (d, 2H, 2CH_Ar_, J = 8.4 Hz); 7.74 (d, 2H, 2CH_Ar_, J = 8.4 Hz); 10.84 (bs, 1H, NH). ^13^C NMR (DMSO-d_6_, 100 MHz); δ (ppm): 26.85 (CH_2_); 27.73 (CH_2_); 33.93 (CH_2_); 38.58 (CH_3_); 39.52 (CH_2_); 59.55 (CH); 60.44 (CH); 93.58 (C); 104.56 (CH); 128.02 (2CH); 134.84 (C); 137.73 (2CH); 149.76 (C); 151.10 (C); 170.45 (C=N). LC-ESI-HRMS (*m/z*) calculated for C_17_H_20_IN_4_S: 439.0454 [M+H]^+^. Found: 439.0456 [M+H]^+^.

##### 4-(4-Methoxyphenyl)-2-(2-(8-methyl-8-azabicyclo[3.2.1]octan-3-ylidene)hydrazinyl)thiazole (**3b**)

Yields: 44%, (dichloromethane/methanol, 80:20, R_f_ = 0.22); mp 91–93 °C. ^1^H NMR (700 MHz, DMSO-d_6_), δ (ppm): 1.29–1.38 (m, 1H, CH_2_); 1.43–1.51 (m, 1H, CH_2_); 1.87–2.00 (m, 2H, 2CH_2_); 2.09 (d, 1H, CH_2_, J = 14.0 Hz); 2.23 (dd, 1H, CH_2_, J = 2.8 Hz, J = 15.2 Hz); 2.29 (s, **3h**, CH_3_); 2.60 (dd, 1H, CH_2_, J = 3.6 Hz, J = 14.8 Hz); 2.68 (d, 1H, CH_2_, J = 15.6 Hz); 3.25 (s, 2H, 2CH); 3.78 (s, **3h**, CH_3_); 6.95 (d, 2H, 2CH_Ar_, J = 9.2 Hz); 7.05 (s, 1H, CH); 7.76 (d, 2H, 2CH_Ar_, J = 9.2 Hz); 10.78 (bs, 1H, NH). ^13^C NMR (DMSO–d_6_, 100 MHz); δ (ppm): 26.62 (CH_2_); 27.47 (CH_2_); 33.80 (CH_2_); 38.51 (CH_3_); 39.56 (CH_2_); 55.51 (OCH_3_); 59.76 (CH); 60.68 (CH); 101.53 (CH); 114.34 (2CH); 127.21 (2CH); 128.15 (C); 149.97 (C); 150.51 (C); 159.09 (C); 170.17 (C=N). LC-ESI-HRMS (*m/z*) calculated for C_18_H_23_N_4_OS: 343.1593 [M+H]^+^. Found: 343.1598 [M+H]^+^.

##### 4-[2-[2-(8-Methyl-8-azabicyclo[3.2.1]oct-3-ylidene)hydrazinyl]-1,3-thiazol-4-yl]benzonitrile (**3c**)

Yields: 26%, (dichloromethane/methanol, 80:20, R_f_ = 0.25); mp 90–92 °C. ^1^H NMR (700 MHz, DMSO-d_6_), δ (ppm): 1.30–1.35 (m, 1H, CH_2_); 1.43–1.48 (m, 1H, CH_2_); 1.88–1.97 (m, 2H, 2CH_2_); 2.09 (d, 1H, CH_2_, J = 14.7 Hz); 2.24 (dd, 1H, CH_2_, J = 3.5 Hz, J = 16.1 Hz); 2.29 (s, **3h**, CH_3_); 2.59 (dd, 1H, CH_2_, J = 3.5 Hz, J = 16.0 Hz); 2.68 (d, 1H, CH_2_, J = 15.4 Hz); 3.25 (s, 2H, 2CH); 7.55 (s, 1H, CH); 7.84 (d, 2H, 2CH_Ar_, J = 8.4 Hz); 8.01 (d, 2H, 2CH_Ar_, J = 8.4 Hz); 10.96 (bs, 1H, NH). ^13^C NMR (DMSO-d_6_, 100 MHz); δ (ppm): 26.84 (CH_2_); 27.72 (CH_2_); 33.56 (CH_2_); 38.56 (CH_3_); 39.52 (CH_2_); 59.57 (CH); 60.45 (CH); 107.61 (CH); 109.84 (C); 119.41 (C=N); 126.46 (2CH); 133.06 (2CH); 139.42 (C); 149.11 (C); 151.43 (C); 170.69 (C=N). LC-ESI-HRMS (*m/z*) calculated for C_18_H_20_N_5_S: 338.1439 [M+H]^+^. Found: 338.1453 [M+H]^+^.

##### 4-(4-Nitrophenyl)-2-(2-(8-methyl-8-azabicyclo[3.2.1]octan-3-ylidene)hydrazinyl)thiazole (**3d**)

Yields: 50%, (dichloromethane/methanol, 80:20, R_f_ = 0.31); mp 99–101 °C. ^1^H NMR (700 MHz, DMSO-d_6_), δ (ppm): 1.29–1.37 (m, 1H, CH_2_); 1.37–1.42 (m, 1H, CH_2_); 1.88–2.00 (m, 2H, 2CH_2_); 2.09 (d, 1H, CH_2_, J = 15.2 Hz); 2.25 (dd, 1H, CH_2_, J = 4.4 Hz, J = 16.6 Hz); 2.29 (s, **3h**, CH_3_); 2.60 (dd, 1H, CH_2_, J = 4.4 Hz, J = 15.2 Hz); 2.68 (d, 1H, CH_2_, J = 16.4 Hz); 3.25 (s, 2H, 2CH); 7.63 (s, 1H, CH); 8.09 (d, 2H, 2CH_Ar_, J = 8.8 Hz); 8.26 (d, 2H, 2CH_Ar_, J = 9.2 Hz); 10.99 (bs, 1H, NH). ^13^C NMR (DMSO-d_6_, 100 MHz); δ (ppm): 26.96 (CH_2_); 27.83 (CH_2_); 34.00 (CH_2_); 38.57 (CH_3_); 39.56 (CH_2_); 59.49 (CH); 60.36 (CH); 108.60 (CH); 126.65 (2CH); 127.47 (2CH); 141.33 (C); 146.48 (C); 148.81 (C); 151.79 (C); 170.79 (C=N). LC-ESI-HRMS (*m/z*) calculated for C_17_H_20_N_5_O_2_S: 358.1338 [M+H]^+^. Found: 358.1347 [M+H]^+^.

##### 4-[2-[2-(8-Methyl-8-azabicyclo[3.2.1]oct-3-ylidene)hydrazinyl]-1,3-thiazol-4-yl]aniline (**3e**)

Yields: 32%, (dichloromethane/methanol, 80:20, R_f_ = 0.14); mp 85–87 °C. ^1^H NMR (700 MHz, DMSO-d_6_), δ (ppm): 1.28–1.35 (m, 1H, CH_2_); 1.41–1.50 (m, 1H, CH_2_); 1.87–1.96 (m, 2H, 2CH_2_); 2.07 (d, 1H, CH_2_, J = 16.8 Hz); 2.19–2.24 (m, 1H, CH_2_); 2.28 (s, **3h**, CH_3_); 2.56–2.60 (m, 1H, CH_2_); 2.63–2.70 (m, 1H, CH_2_); 3.24 (s, 2H, 2CH); 5.17 (bs, 2H, NH_2_); 6.53 (d, 2H, 2CH, J = 8.4 Hz); 6.78 (s, 1H, CH); 7.48 (d, 2H, 2CH_Ar_, J = 8.4 Hz); 10.68 (bs, 1H, NH). ^13^C NMR (DMSO-d_6_, 100 MHz); δ (ppm): 26.05 (CH_2_); 26.83 (CH_2_); 33.45 (CH_2_); 38.22 (CH_3_); 39.60 (CH_2_); 60.24 (CH); 61.23 (CH); 98.82 (CH); 112.91 (CH); 114.07 (2CH); 126.91 (2CH); 131.86 (C); 148.68 (C); 154.23 (C); 169.77 (C=N). LC-ESI-HRMS (*m/z*) calculated for C_17_H_22_N_5_S: 328.1596 [M+H]^+^. Found: 328.1608 [M+H]^+^.

##### *N*-(4-[2-[2-(8-Methyl-8-azabicyclo[3.2.1]oct-3-ylidene)hydrazinyl]-1,3-thiazol-4-yl]phenyl)methane-sulfonamide (**3f**)

Yields: 30%, (dichloromethane/methanol, 80:20, R_f_ = 0.18); mp 98–100 °C. ^1^H NMR (700 MHz, DMSO-d_6_), δ (ppm): 1.30–1.35 (m, 1H, CH_2_); 1.43–1.48 (m, 1H, CH_2_); 1.88–1.96 (m, 2H, 2CH_2_); 2.08 (d, 1H, CH_2_, J = 14.0 Hz); 2.21–2.25 (m, 1H, CH_2_); 2.29 (s, **3h**, CH_3_); 2.56–2.61 (m, 1H, CH_2_); 2.68 (d, 1H, CH_2_, J = 14.0 Hz); 3.00 (s, **3h**, CH_3_); 3.24 (s, 2H, 2CH); 7.13 (s, 1H, CH); 7.21 (d, 2H, 2CH, J = 8.4 Hz); 7.78 (d, 2H, 2CH_Ar_, J = 8.4 Hz); 9.79 (bs, 1H, NH); 10.82 (bs, 1H, NH). ^13^C NMR (DMSO-d_6_, 100 MHz); δ (ppm): 26.72 (CH_2_); 27.58 (CH_2_); 28.00 (CH_3_); 33.87 (CH_2_); 38.54 (CH_3_); 39.07 (CH_2_); 59.67 (CH); 60.58 (CH); 102.86 (CH); 120.03 (2CH); 126.92 (2CH); 131.05 (C); 137.94 (C); 150.26 (C); 150.45 (C); 170.38 (C=N). LC-ESI-HRMS (*m/z*) calculated for C_18_H_24_N_5_O_2_S_2_: 406.1371 [M+H]^+^. Found: 406.1375 [M+H]^+^.

##### 3-[2-[2-(8-Methyl-8-azabicyclo[3.2.1]oct-3-ylidene)hydrazinyl]-1,3-thiazol-4-yl]-2H-chromen-2-one (**3g**)

Yields: 44%, (dichloromethane/methanol, 80:20, R_f_ = 0.44); mp 110–113 °C. ^1^H NMR (700 MHz, DMSO-d_6_), δ (ppm): 1.32–1.39 (m, 1H, CH_2_); 1.45–1.52 (m, 1H, CH_2_); 1.90–1.99 (m, 2H, 2CH_2_); 2.12 (d, 1H, CH_2_, J = 15.4 Hz); 2.28 (d, 1H, CH_2_, J = 16.8 Hz); 2.32 (s, **3h**, CH_3_); 2.63 (d, 1H, CH_2_, J = 15.4 Hz); 2.69 (d, 1H, CH_2_, J = 14.0 Hz); 3.29 (s, 2H, 2CH); 7.38 (t, 1H, CH, J = 6.3 Hz); 7.45 (d, 1H, CH, J = 7.0 Hz); 7.60–7.63 (m, 1H, CH); 7.69 (s, 1H, CH); 7.81 (dd, 1H, CH, J = 1.4 Hz, J = 7.7 Hz); 8.53 (s, 1H, CH); 10.93 (bs, 1H, NH). ^13^C NMR (DMSO-d_6_, 100 MHz); δ (ppm): 26.77 (CH_2_); 27.64 (CH_2_); 33.89 (CH_2_); 38.50 (CH_3_); 39.59 (CH_2_); 59.64 (CH); 60.54 (CH); 110.75 (CH); 116.28 (C); 119.61 (C); 121.13 (C); 125.14 (C); 129.12 (C); 132.05 (C); 138.34 (C); 144.24 (C); 151.11 (C); 152.66 (C); 159.14 (C); 169.81 (C=N). LC-ESI-HRMS (*m/z*) calculated for C_20_H_21_N_4_O_2_S: 381.1385 [M+H]^+^. Found: 381.1390 [M+H]^+^.

##### *N*-(4-[2-[2-(8-Methyl-8-azabicyclo[3.2.1]oct-3-ylidene)hydrazinyl]-1,3-thiazol-4-yl]phenyl)acetamide (**3h**)

Yields: 26%, (dichloromethane/methanol, 80:20, R_f_ = 0.25); mp 97–99 °C. ^1^H NMR (700 MHz, DMSO-d_6_), δ (ppm): 1.30–1.36 (m, 1H, CH_2_); 1.44–1.50 (m, 1H, CH_2_); 1.89–1.97 (m, 2H, 2CH_2_); 2.04 (s, **3h**, CH_3_); 2.07–2.11 (m, 1H, CH_2_); 2.22–2.26 (m, 1H, CH_2_); 2.30 (s, **3h**, CH_3_); 2.57–2.63 (m, 1H, CH_2_); 2.66–2.71 (m, 1H, CH_2_); 3.24 (s, 2H, 2CH); 7.08 (s, 1H, CH); 7.58 (d, 2H, 2CH, J = 9.1 Hz); 7.74 (d, 2H, 2CH, J = 8.5 Hz); 9.98 (bs, 1H, NH); 10.81 (bs, 1H, NH). ^13^C NMR (DMSO-d_6_, 100 MHz); δ (ppm): 24.42 (CH_3_); 26.83 (CH_2_); 27.70 (CH_2_); 33.90 (CH_2_); 38.56 (CH_3_); 39.51 (CH_2_); 59.58 (CH); 60.48 (CH); 102.28 (CH); 119.30 (2CH); 126.27 (2CH); 130.23 (C); 139.01 (C); 150.56 (C); 150.68 (C); 168.65 (C = O); 170.27 (C=N). LC-ESI-HRMS (*m/z*) calculated for C_19_H_24_N_5_OS: 370.1702 [M+H]^+^. Found: 370.1707 [M+H]^+^.

### 3.2. In Vitro Experiments

#### 3.2.1. Cell Cultures and Drug Treatment

The human multiple myeloma (MM) RPMI 8226 cell line was purchased from the European Collection of Authenticated Cell Cultures (ECACC). The human Caucasian non-small-cell lung carcinoma A549 cell line and the human normal colon fibroblast CCD-18Co cell line were purchased from the American Type Culture Collection (ATCC, Manassas, VA, USA). The human normal skin fibroblasts, i.e., the HSF cell line, were a laboratory strain obtained with the outgrowth technique from skin explants from young volunteers (with subjects’ written informed consent). Mouse melanoma B16F10 and human breast adenocarcinoma MDA-MB-231 cells were kindly gifted by Prof. Tomasz Drewa, a Chair of Urology, Department of Regenerative Medicine, Collegium Medicum, Nicolaus Copernicus University, Bydgoszcz, Poland). The RPMI 8226 cells were cultured in RPMI 1640 medium (Sigma-Aldrich Chemicals, St. Louis, MO, USA) supplemented with penicillin (100 units/mL, Sigma, St. Louis, MO, USA) and streptomycin (100 μg/mL, Sigma-Aldrich) and 10% fetal bovine serum (FBS; *v*/*v*, Sigma-Aldrich, St. Louis, MO, USA). The A549 adenocarcinoma cells were grown in a 2:1 mixture of Dulbecco’s modified Eagle Medium and Ham’s F12 Medium (Sigma-Aldrich) supplemented with 10% FBS, 100 units/mL of penicillin, and 100 of µg/mL streptomycin. The CCD18-Co cells were grown in Eagle Medium (Sigma-Aldrich, St. Louis, MO, USA) supplemented with 2 mM L-glutamine (Sigma-Aldrich) and 1mM sodium pyruvate (Sigma-Aldrich, St. Louis, MO, USA), 10% FBS, (without antibiotics). HSF were cultured in a 1:1 mixture of Dulbecco’s modified Eagle Medium and Eagle Medium (Sigma-Aldrich) containing 10% FBS, 100 units/mL of penicillin, and 100 mg/mL of streptomycin. B16F10 and MDA-MB-231 cell lines were cultured in RPMI cell culture medium (with l-glutamine and sodium bicarbonate, Sigma-Aldrich) supplemented with 10% of fetal bovine serum (FBS, Biowest, Nuaillé, France), as well as antibiotics and antimycotics (Sigma-Aldrich). All cell lines were cultured in a humidified 5% CO_2_ atmosphere at a temperature of 37 °C. A stock solution of each tested compound was made in dimethylsulfoxide (DMSO; Sigma-Aldrich) and stored at 4 °C until use. The drug was diluted in an appropriate culture medium immediately before use. The DMSO concentration did not exceed 0.1% *v*/*v*.

#### 3.2.2. Cell Proliferation Assay

The influence of the tested compounds on the proliferation of RPMI 8226 and A549 cells was estimated with the MTT assay as described previously [32]. Briefly, 1 × 10^4^ cells/mL (RPMI 8226) or 0.2 × 10^4^ (A549) cells per well were seeded into 96-well plates and cultured (in growth medium containing 10% FBS) with different concentrations of each compound ranging from 3.125 to 100 μM. The control cells were treated with the vehicle DMSO (0.1%). After 96 h exposure, the cells were incubated for 3 h with an MTT solution (5 mg/mL, Sigma-Aldrich) and then formazan crystals were solubilized overnight by adding SDS buffer (10% SDS in 0.01 N HCl). Absorbance was determined at a wavelength of 570 nm using an EL800 Microplate Reader (BioTek Instruments, Winooski, VT, USA). The absorbance of the control wells was taken as 100% and the results were expressed as a percentage of the control. IC_50_ values, defined as concentrations of the drugs necessary to inhibit 50% of growth compared to untreated control cells, were calculated from nonlinear regression (log(inhibitor) vs. normalized response-variable slope) using GraphPad Prism 5.0.

The influence of the tested compounds on the proliferation of melanoma B16F10 and breast MDA-MB-231 cells was estimated with the MTT assay as described previously [14]. The cells counted using an automated cell counter (Countess^®^ II FL, Invitrogen by Thermo Fisher Scientific Inc., Waltham, MA USA) and plated in 96-well plates (Nunc Edge 2F, Thermo Fisher Scientific Inc.) at a density of 0.1 × 10^4^ and 0.2 × 10^4^ per well, respectively. A total of twenty-four hours later, drugs were applied with final concentration: 5, 0.5, 0.05, and 0.005 μg/mL, and after an additional seventy-two hours MTT staining was performed as follows: twenty microliters of 5 mg/mL MTT solution and was added to each well of 96-well plate incubated for 2 h. Subsequently, the medium was removed and crystals of formazan were solubilized in pure isopropanol. Absorbance readout was conducted immediately using plate reader Multiskan Spectrum at 570 nm (Thermo Electron Corporation, Waltham, MA, USA). The background optical density was measured in the wells filled with culture medium, without the cells. The biological evaluation was performed as three independent experiments. The IC_50_ values were calculated using ED50plus v 1.0 freeware software and the mean response and standard deviation were calculated using Excel software. All of the chemicals were obtained from Sigma-Aldrich.

#### 3.2.3. Cell Cytotoxicity Assay

The influence of the tested compounds on normal cell viability was estimated with the MTT assay. The CCD-18Co and HSF cells were seeded in 96-well microplates at a density of 1 × 10^5^ cells/mL in complete growth medium. The next day, the medium was replaced with a fresh one containing 2% FBS (growth restriction) and the cells were exposed to each tested compound (3.156–100 µM) to study the acute toxicity in vitro. After 24-h incubation, the medium was removed and the cells were incubated for 3 h with an MTT solution; next, formazan crystals were solubilized overnight by adding SDS buffer and absorbance was determined at a wavelength of 570 nm using an EL800 Microplate Reader (BioTek Instruments, Winooski, VT, USA). The absorbance of the control wells was taken as 100% and the cell viability was expressed as a percentage of the control. CC_50_ values, defined as concentrations of the compounds necessary to reduce cell viability by 50%, were calculated from nonlinear regression (log(inhibitor) vs. normalized response-variable slope) using GraphPad Prism 5.0.

#### 3.2.4. Cell Cycle Progression Assay

The RPMI 8226 or A549 cells were seeded in 6-well plates in medium containing 10% FBS without or with compound **3a** (2.5, 5, 10, 20, or 40 µM). After 24-h and 48-h treatment, the cell cycle analysis was performed by determination of the DNA contents on the basis of PI staining as described previously [33]. Briefly, the cells were harvested, washed with PBS, and fixed overnight with 80% ethanol at −20 °C. After that, the cells were washed with PBS and stained with PI using PI/RNase Staining Buffer (BD Biosciences, BD Pharmingen™, USA) for 30 min in darkness at RT. Next, the stained cells were analyzed using FACS Calibur. The intensity of PI fluorescence of individual nuclei was determined and at least 10 000 events were measured within an acquisition rate >60 events/s. The FACS data were analyzed using Cell Quest Pro Version 6.0 (BD Biosciences, San Jose, CA, USA). for the Macintosh operating system. The data were analyzed to determine the percentage of cells at the sub-G1 phase (apoptotic cells) and each phase of the cell cycle (G1, S, and G2/M).

#### 3.2.5. Evaluation of Cell Death by Flow Cytometry

The quantitative analysis of compound **3a**-induced cell death was performed using an Annexin V-fluorescein isothiocyanate (FITC)/propidium iodide (PI) apoptosis kit (BD Biosciences, BD Pharmingen™, San Jose, CA, USA) as previously described [32]. Briefly, the RPMI 8226 or A549 cells growing on the 6-well plates in the culture medium with 2% FBS were subjected to increasing concentrations of compound **3a** (2.5, 5, 10, 20, or 40 μM) for 24 and 48 h. After the treatment, the cells were harvested, centrifuged at 1000 rpm and room temperature (RT) for 5 min, washed twice with PBS, and stained with 5 mM of AnnexinV-FITC and 5 mM of PI. After incubation for 15 min in the dark at room temperature, the cells were immediately analyzed using FACS Calibur. The intensity of fluorescence of AnnexinV-FITC and PI stained cells was determined by acquisition of at least 10,000 events per sample within the rate >60 events/s. The FACS data were analyzed using Cell Quest Pro Version 6.0. for the Macintosh operating system. The data were analyzed to determine the percentage of viable, early apoptotic, late apoptotic, and necrotic cells.

#### 3.2.6. Statistical Analysis

Statistical analysis was performed by one-way ANOVA followed by Dunnett’s or Tukey’s multiple comparison test. All the results were presented as the mean ± standard deviation (SD), (* *p* < 0.05, ** *p* < 0.01, *** *p* < 0.001).

### 3.3. Mushroom Tyrosinase Inhibition Assay

The mushroom tyrosinase (Sigma-Aldrich) inhibition was performed following previously reported methods [34]. All the assays were carried out with solutions containing phosphate buffer (50 mM, pH 6.8), L-DOPA (0.17 mM), EDTA (0.022 mM), tyrosinase (50–100 units) and varying concentrations of compounds and were done in triplicate at room temperature. The inhibitor solutions were prepared in DMSO with an initial concentration of 1 mM. Different aliquots were added to the solution containing buffer, L-DOPA and EDTA, the enzyme being added in the end. Formation of dopachromone was determined by monitoring the absorbance at 475 nm with a T60U spectrophotometer (PG Instruments, Leicestershire, UK) equipped with quartz cells of 1 cm path length. Ascorbic acid was used as a reference inhibitor with an initial concentration of 1 mM. The IC_50_ values were calculated from the equation generated by exponential fit of the experimental data. The effectiveness of inhibition was expressed for the investigated compounds as the percentage of concentration necessary to achieve 50% inhibition (IC_50_), calculated using the following equation:

% of Inhibition = {[(B30 − B0) − (A30 − A0)]/(B30 − B0)} × 100

where B0 = absorbance of L-DOPA + tyrosinase at t = 0 min, B30 = absorbance of L-DOPA + tyrosinase at t = 30 min, A0 = absorbance of L-DOPA + tyrosinase + inhibitor at t = 0 min, and A30 = absorbance of L-DOPA + tyrosinase + inhibitor at t = 30 min.

#### Kinetic Analysis of the Inhibition of Tyrosinase

A series of experiments were performed to determine the inhibition kinetics of compounds by following the already reported method [35]. The inhibitor concentrations for compounds were 50 and 100 µM. Substrate L-DOPA concentration was between 100 and 250 µM in all kinetic studies. Maximal initial velocity was determined from the initial linear portion of absorbance up to ten minutes after addition of enzyme. The inhibition type of the enzyme, Michaelis constant (K_M_) and maximal velocity (V_max_) were determined by Lineweaver–Burk plots of inverse of velocities (1/V) versus inverse of substrate concentration 1/[L-DOPA] mM^−1^.

### 3.4. Docking Study

The ligand molecules were prepared by using the Avogadro 1.1.1 software [36] and optimized within the UFF force field [37] (5000 steps, steepest descent algorithm). The flexible, optimized ligands were docked into the binding pocket of the protein structure found in the PDB database: 2y9x (X-ray resolution: 2.78 Å). The PDB record contains four different structures of the same protein; all of them were considered during the docking procedure. The AutoDock Vina software [38] was applied for docking simulations. The procedure of docking was carried out within the cuboid region of dimensions of 18 × 18 × 18 Å^3^, which covers all the originally co-crystallized ligands present in the PDB structures as well as the closest amino-acid residues that exhibit contact with those ligands. All the defaults procedures and algorithms implemented in AutoDock Vina were applied during docking procedure. The predicted binding energies were averaged over all of the four protein structures. The more favorable binding mode is associated with the lower binding energy value; only the lowest energy values and ligand poses associated with them were accounted for during subsequent analysis.

The docking methodology was initially validated by docking simulations of the ligand molecule originally included in the protein structure. The description of the validation procedure and the graphical illustration of its results can be found in [14]. Here, we only mention that the accepted methodology is accurate enough to recover the original position of the bound ligand.

### 3.5. Quantum Calculation

In the present work, quantum mechanical studies were performed for **3a**–**3h** molecules. As a first step, two isomers of each of the investigated compounds **3a**–**3h** were built (see Scheme S1), and their geometrical parameters were optimized employing the DFT method. The B3LYP exchange-correlation functional and the 6-311G** basis set were used for that purpose, based on our earlier findings. In the case of **3a**, the LanL2DZ basis set was used on iodine atom. Optimizations were followed by vibrational frequencies evaluation carried out at the same level of approximation to confirm that optimized structures correspond to real minima on potential energy surface. Of each pair of isomers, only the lower energy isomer, constituting practically 100% of the population, was investigated further. Isomer fraction populations calculated as in reference [39] are presented in Supplementary Material (Table S6). The MEP surfaces were obtained based on the B3LYP/6-311G** calculations using the Gaussian 09 package [40], and next visualized with GaussView 5 [41]. In the MEP surface figure, blue colour denotes the most positive electronic potential, which is most electrophilic regions, while red colour denotes those with most negative electronic potential, which is most nucleophilic regions. Thus, the parts of the surface denoted with red colour are electron-rich, and those blue—electron-deficient. Cartesian coordinates of the resulting lower-energy structures are presented in the Supplementary Material.

## 4. Conclusions

Using filters such as the Lipinski and Veber rules, as well as performing quantum calculations, we managed to design and then synthesize with good efficiency eight tropinone-derived alkaloids. All derivatives **3a**–**3h** showed high antitumor activity against human breast adenocarcinoma (MDA-MB-231) and mouse skin melanoma (B16-F10) cell lines with IC_50_ values of 1.51–3.03 µM, and showed negligible toxicity against the human normal HSF and CCD-18Co cells. We have successfully identified iodo-derivative **3a** to show high activity against human multiple myeloma (RPMI 8226) with an IC_50_ value of 9.32 µM. Its mechanism of action is to induce cell death through apoptosis. The most active compounds **3e** and **3h** showed tyrosinase inhibition effect, with IC_50_ values approximately 120 µM, and detailed molecular docking showed distinct correlation with −log(IC_50_) values. Overall, our research has shown that tropinone derivatives have high potential for a new generation of anticancer drugs.

## Figures and Tables

**Table 1:** Predicted molecular properties for compounds **3a**–**3h**.

	Lipiński’s Rules				Veber’s Rules		Quantum Parameters							
Tropinone	*logP*	HBA	HBD	MW	NRB	TPSA	*E_HOMO_*	*E_LUM__O_*	Δ*E*	*η*	*S*	*IP*	*EA*	*χ*
**3a**	3.87	3	1	438.33	3	68.76	−5.490	−1.189	4.301	2.150	0.465	5.490	1.189	3.340
**3b**	3.23	4	1	342.46	4	77.99	−5.142	−0.836	4.306	2.153	0.464	5.142	0.836	2.989
**3c**	3.30	4	1	337.44	3	92.55	−5.734	−1.791	3.943	1.971	0.507	5.734	1.791	3.762
**3d**	2.58	5	1	357.43	4	114.58	−5.804	−2.454	3.350	1.675	0.597	5.804	2.454	4.129
**3e**	2.66	3	2	327.45	3	94.78	−4.934	−0.733	4.201	2.101	0.476	4.934	0.733	2.833
**3f**	2.41	5	2	405.54	5	123.31	−5.463	−1.188	4.274	2.137	0.468	5.463	1.188	3.326
**3g**	3.28	5	1	380.46	3	98.97	−5.492	−2.006	3.487	1.743	0.574	5.492	2.006	3.749
**3h**	2.81	4	2	369.48	5	97.86	−5.223	−0.993	4.230	2.115	0.473	5.223	0.993	3.108

**Table 2:** Antiproliferative activity of compounds **3a**–**3h** against human multiple myeloma (RPMI 8226), human lung carcinoma (A549), human breast adenocarcinoma (MDA-MB-231), and mouse skin melanoma (B16-F10) cell lines. Effect of compounds **3a**–**3h** on viability of human normal skin fibroblasts (HSF) and human normal colon fibroblasts (CCD-18Co).

Tropinone Derivatives	IC_50_ ± SD (µM)				CC_50_ ± SD (µM)	
	RPMI 8226	A549	MDA-MB-231	B16-F10	HSF	CCD-18Co
**3a**	9.32 ± 0.71	13.81 ± 1.90	2.26 ± 0.38	1.51 ± 0.14	19.23 ± 1.77	27.76 ± 8.85
**3b**	42.19 ± 4.55	132.7 ± 25.12	2.53 ± 0.07	2.20 ± 0.04	51.46 ± 12.85	92.60 ± 1.69
**3c**	31.70 ± 2.77	74.93 ± 7.65	2.70 ± 0.14	1.52 ± 0.13	78.12 ± 1.05	101.28 ± 2.61
**3d**	17.87 ± 1.78	30.99 ± 4.62	2.44 ± 0.14	1.91 ± 0.04	35.36 ± 0.97	45.57 ± 3.12
**3e**	67.03 ± 7.14	262.9 ± 104.9	2.81 ± 0.12	2.68 ± 0.40	nt	nt
**3f**	107.3 ± 7.22	117.1 ± 22.82	2.48 ± 0.08	2.14 ± 0.23	98.67 ± 4.16	nt
**3g**	23.16 ± 4.52	29.29 ± 3.98	2.83 ± 0.06	3.00 ± 0.26	35.29 ± 5.52	42.58 ± 5.89
**3h**	107.9 ± 4.80	188.8 ± 33.02	2.90 ± 0.25	3.03 ± 0.18	nt	nt
Chlorambucil	-	-	4.71 ± 0.5	2.92 ± 0.10	-	-

nt—non-toxic in the range of the tested concentrations (3.125–100 µM).

**Table 3:** Tyrosinase inhibitory activity of tropinone-thiazole derivatives **3a**–**3h** compared to standard inhibitor ascorbic acid. The mechanism of inhibition, maximum velocity (V_max_), and inhibition constant (K_M_) in the tyrosinase-catalyzed oxidation of L-DOPA. Compounds at concentration 100 µM.

Compound	IC_50_ ± SD (µM)	Inhibitory Mechanism	V_max_	K_M_
**3a**	151.44 ± 6.80	mixed	−325.9	−515.6
**3b**	165.38 ± 3.47	mixed	−280.2	−409.1
**3c**	153.04 ± 7.42	mixed	−753.9	−914.4
**3d**	140.21 ± 7.96	mixed	4601.9	4206.2
**3e**	118.06 ± 1.77	mixed	3392.1	3191.3
**3f**	152.04 ± 4.66	mixed	−350.2	−536.4
**3g**	183.34 ± 6.18	mixed	−4472.3	−3295.2
**3h**	120.71 ± 5.21	mixed	−271.8	−433.2
Ascorbic acid	386.5 ± 10.34	-	-	-

**Table 4:** Binding energies recovered during docking with respect to the studied ligands. The calculation results were averaged over four different structures of the same 2y9x protein available in the same PDB record; the corresponding standard deviations are given.

Compound	Binding Energy ± SD (kcal/mol)
**3a**	−5.8 ± 0.2
**3b**	−6.4 ± 0.2
**3c**	−7.5 ± 0.3
**3d**	−7.2 ± 0.2
**3e**	−6.5 ± 0.2
**3f**	−6.3 ± 0.1
**3g**	−5.4 ± 0.1
**3h**	−8.1 ± 0.2
Ascorbic acid	−4.4 ± 0.6 *

* the value additionally averaged over three tautomeric forms of ascorbate ion.

## Supplementary Materials

The following are available online at https://www.mdpi.com/1422-0067/21/23/9050/s1, Table S1: Pearson’s correlation matrix for descriptors collected in Table 1 and the experimental pIC50 = −log (IC50) values, Tables S2 and S3: Effect of compound **3a** on cell cycle distribution in the RPMI 8226 and A549 cell lines cultures, Tables S4 and S5: Effects of compound **3a** on apoptosis (early + late apoptosis) induction in the RPMI 8226 and A549 cells, ^1^H, ^13^C NMR and LC-ESI-HRMS spectra of compounds **3a**–**3h**, Molecular orbital distribution plots of HOMO-LUMO for compound **3a**–**3h** at B3LYP/6-311G** level, Geometrical parameters of the investigated compounds **3a**–**3h** at B3LYP/6-311G** level, Scheme S1: Structure of isomers 1 and 2 of investigated compounds, Table S6: Fractional populations [%] of isomers 1 and 2 of investigated compounds.
