# Supplementary material for: Tropinone-Derived Alkaloids as Potent Anticancer Agents: Synthesis, Tyrosinase Inhibition, Mechanism of Action, DFT Calculation, and Molecular Docking Studies
Source: Int J Mol Sci. 2020 Nov 28;21(23):9050. doi: 10.3390/ijms21239050 (PMC7731314; doi:10.3390/ijms21239050)
Supplement: Supplementary file 1 [file ijms-21-09050-s001.pdf]

Supplementary material

1. Table S1. Pearson's correlation matrix for descriptors collected in Table 1 and the experimental pIC_{50} = -log (IC_{50}) values.

2. Table S2-S3. Effect of compound **3a** on cell cycle distribution in the RPMI 8226 and A549 cell lines cultures.

3. Table S4-S5. Effects of compound **3a** on apoptosis (early + late apoptosis) induction in the RPMI 8226 and A549 cells.

4. ^{1}H, ^{13}C NMR and LC-ESI-HRMS spectra of compounds **3a**-**3h**.

5. Molecular orbital distribution plots of HOMO-LUMO for compound **3a**-**3h** at B3LYP/ 6-311G** level

6. Geometrical parameters of the investigated compounds **3a**-**3h** at B3LYP/6-311G** level.

7. Scheme S1. Structure of isomers 1 and 2 of investigated compounds.

8. Table S6. Fractional populations [%] of isomers 1 and 2 of investigated compounds.

Table S1. Pearson's correlation matrix for descriptors collected in Table 1 and the experimental pIC_{50} = -log (IC_{50}) values.

	log*P*	HBA	HBD	MW	NRB	TPSA	E_{HOMO}	E_{LUMO}	ΔE	η	S	IP	EA	χ	pIC_{50}
**log*P***	1.000														
**HBA**	-0.450	1.000													
**HBD**	-0.666	-0.124	1.000												
**MW**	0.355	0.036	-0.052	1.000											
**NRB**	-0.615	0.435	0.545	0.133	1.000										
**TPSA**	-0.864	0.749	0.432	-0.094	0.532	1.000									
**E_{HOMO}**	-0.128	-0.536	0.571	-0.264	0.078	-0.295	1.000								
**E_{LUMO}**	0.019	-0.648	0.575	-0.016	0.194	-0.397	0.872	1.000							
**ΔE**	0.129	-0.623	0.480	0.177	0.249	-0.408	0.626	0.928	1.000						
**η**	0.128	-0.623	0.481	0.176	0.249	-0.408	0.627	0.928	1.000	1.000					
**S**	-0.138	0.629	-0.479	-0.156	-0.228	0.412	-0.622	-0.925	-0.999	-0.999	1.000				
**IP**	0.128	0.536	-0.571	0.264	-0.078	0.295	-1.000	-0.872	-0.626	-0.627	0.622	1.000			
**EA**	-0.019	0.648	-0.575	0.016	-0.194	0.397	-0.872	-1.000	-0.928	-0.928	0.925	0.872	1.000		
**χ**	0.030	0.630	-0.591	0.100	-0.161	0.375	-0.940	-0.987	-0.854	-0.855	0.851	0.940	0.987	1.000	
**pIC_{50}**	-0.472	-0.459	0.694	-0.282	0.260	0.134	0.410	0.384	0.298	0.299	-0.295	-0.410	-0.384	-0.404	1.000

Table S2. Effect of compound **3a** on cell cycle distribution in the RPMI 8226 cell line culture. After the treatment with various concentrations of compound 1 for 24 and 48 h, the cells were stained with propidium iodide and analyzed by flow cytometry. **p* < 0.05, ***p* < 0.01, and *p* < 0.001*** in comparison to the control; one-way ANOVA test

Concentration (μM)	24 h				48 h			
	Cell cycle distribution [% of cells]							
	Sub-G1	G0/G1	S	G2	Sub-G1	G0/G1	S	G2
0	11.56±5.5	41.93±6.15	26.36±2.04	20.35±3.78	8.6±2.85	40.57±1.8	28.8±1.53	22.71±1.92
2.5	15.53±2.23	41.14±1.03	21.59±1.77	22.35±1.19	21.49±0.94**	35.8±1.49	26.22±0.97	16.99±2
5	21.32±6.2*	38.23±6.83	18.68±2.58	21.31±4.02	25.53±7.59***	40.3±3.15	19.12±2.51	15.23±5.04
10	23.53±7.04**	38.08±9.38	21.4±3.5	17.45±2.75	28.01±5.09***	38.5±2.77	18.61±4.5	18.78±2.32
20	24.09±2.62**	42.63±4.03	19.83±2.92	15.7±5.79	29.68±10.49***	31.66±10.94	20.74±2.14	17.47±1.42

Table S3. Effect of compound **3a** on cell cycle distribution in the A549 cell line culture. After the treatment with various concentrations of compound 1 for 24 and 48 h, the cells were stained with propidium iodide and analyzed by flow cytometry. **p* < 0.05, and *p* < 0.001*** in comparison to the control; one-way ANOVA test

Concentration (μM)	24 h				48 h			
	Cell cycle distribution [% of cells]							
	sub-G1	G0/G1	S	G2/M	sub-G1	G0/G1	S	G2/M
0	8.29±4.27	64.55±2.73	13.13±2.69	14.49±2.12	13.81±4.87	70.70±6.85	7.22±2.75	8.41±1.53
2.5	8.81±3.73	64.49±1.89	12.39±2.34	14.68±2.11	11.98±3.62	73.22±1.97	6.07±1.54	8.91±2.22
5	8.94±3.39	65.90±2.36	12.18±2.45	13.62±2.25	15.74±6.03	68.55±8.03	6.81±2.66	9.05±1.72
10	13.23±7.01	66.51±4.46	9.82±2.73	11.43±2.88	16.15±6.33	68.54±6.87	7.07±2.63	8.44±1.47
20	22.36±12.24***	66.45±9.20	5.23±2.38*	7.16±2.15	27.32±6.78***	63.07±6.59*	4.61±1.21	5.19±0.93
40	35.46±9.33***	41.03±2.93***	11.34±0.58	12.87±5.29	75.27±4.12***	16.49±3.46***	6.05±0.87	2.19±0.38

Table S4. Effects of compound **3a** on apoptosis (early + late apoptosis) induction in the RPMI 8226 cells. After the 24-h and 48-h exposure, the cells were stained with AnnexinV-FITC/propidium iodide and examined with flow cytometry. Statistically significant at *p* < 0.01** or at *p* < 0.001*** in comparison to the vehicle control; one-way ANOVA test.

Concentration [μM]	24 hours		48 hours	
	Apoptosis [% of cells ± SD]	Necrosis [% of cells ± SD]	Apoptosis [% of cells ± SD]	Necrosis [% of cells ± SD]
0	14.33 ± 0.87	5.03 ± 0.86	12.99 ± 2.44	5.48 ± 2.25
2.5	19.53 ± 2.64**	3.62 ± 0.87	20.93 ± 1.83***	5.54 ± 0.44
5	22.23 ± 6.01**	6.35 ± 2.57	31.71 ± 11.36***	7.53 ± 2.61
10	39.58 ± 9.33***	9.94 ± 2.73	58.25 ± 21.57***	11.08 ± 5.38
20	60.41 ± 0.99***	18.5 ± 2.38***	76.93 ± 14.34***	20.18 ± 11.99***

Table S5. Effects of compound **3a** on apoptosis (early + late apoptosis) induction in the A549 cells. After the 24-h and 48-h exposure, the cells were stained with AnnexinV-FITC/propidium iodide and examined with flow cytometry. Statistically significant at $p < 0.01$** or at $p < 0.001$*** in comparison to the vehicle control; one-way ANOVA test.

Concentration [μM]	24 hours		48 hours	
	Apoptosis [% of cells ± SD]	Necrosis [% of cells ± SD]	Apoptosis [% of cells ± SD]	Necrosis [% of cells ± SD]
0	14.14±1.77	4.72±1.29	13.65±3.71	5.01±2.20
2.5	14.30±2.21	4.79±1.14	18.41±3.95	4.11±1.44
5	14.82±2.42	4.40±1.66	22.48±8.36	4.93±4.26
10	18.18±2.52	4.87±1.35	27.17±11.71**	4.09±2.58
20	23.43±4.57**	4.56±1.90	40.20±9.87***	4.87±3.04
40	73.96±19.94***	7.89±1.10	89.43±6.65***	8.81±6.51

3a

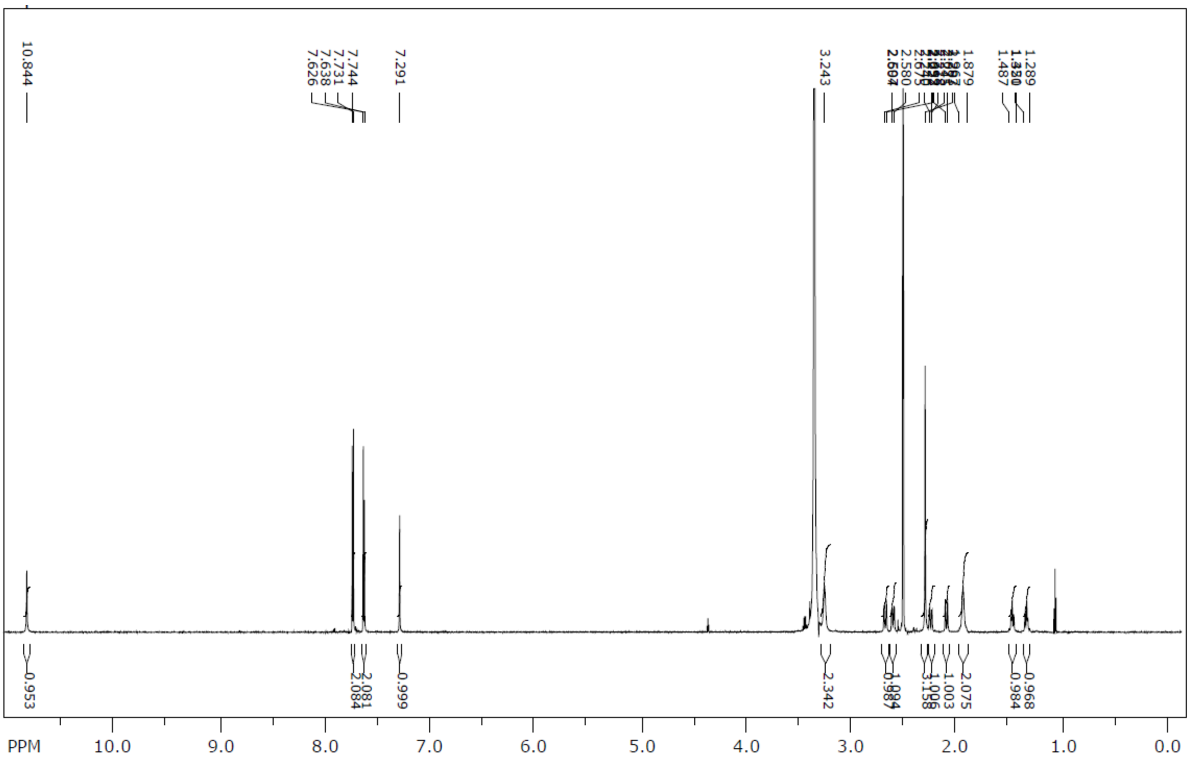

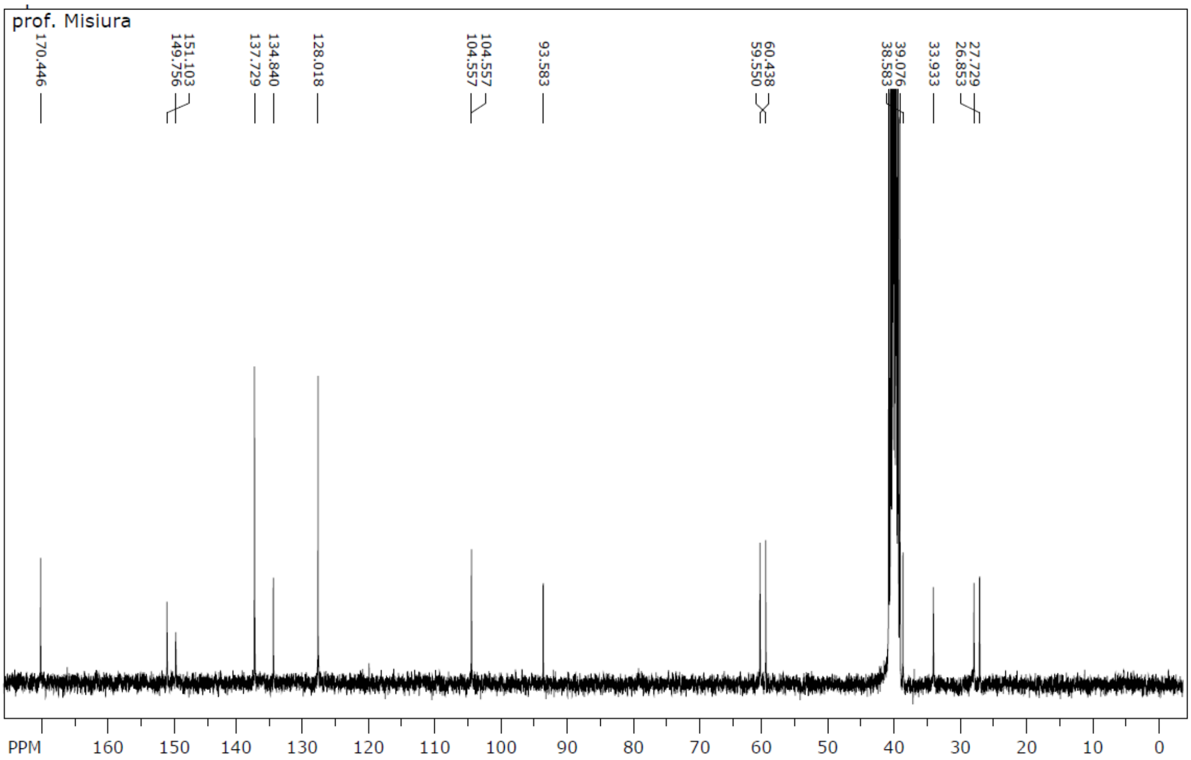

prof. Misiura
170.446
151.103
149.756
137.729
134.840
128.018
104.557
104.557
93.583
60.438
59.550
39.076
38.583
33.933
27.729
26.853
PPM 160 150 140 130 120 110 100 90 80 70 60 50 40 30 20 10 0

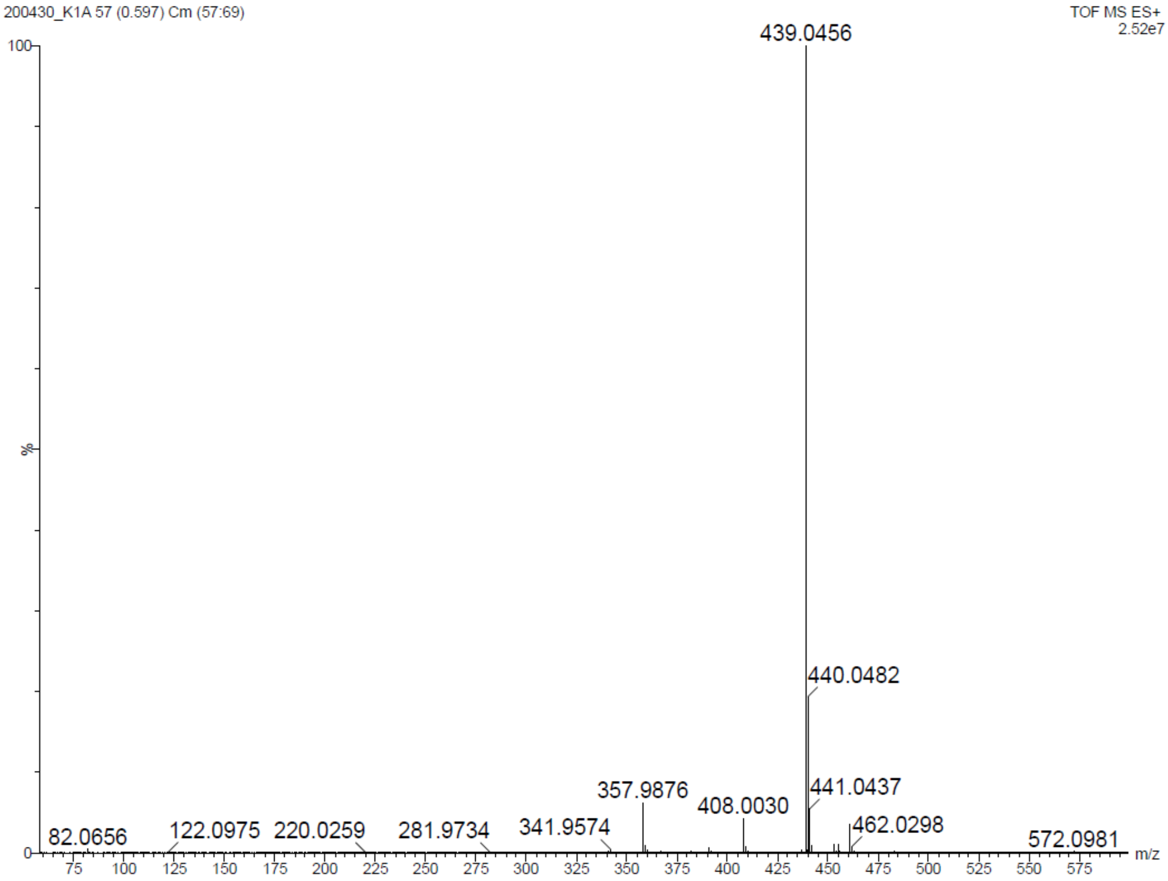

200430_K1A 57 (0.597) Cm (57:69)
TOF MS ES+
2.52e7
100
%
0
439.0456
440.0482
441.0437
462.0298
408.0030
357.9876
341.9574
281.9734
220.0259
122.0975
82.0656
572.0981
75 100 125 150 175 200 225 250 275 300 325 350 375 400 425 450 475 500 525 550 575 m/z

3b

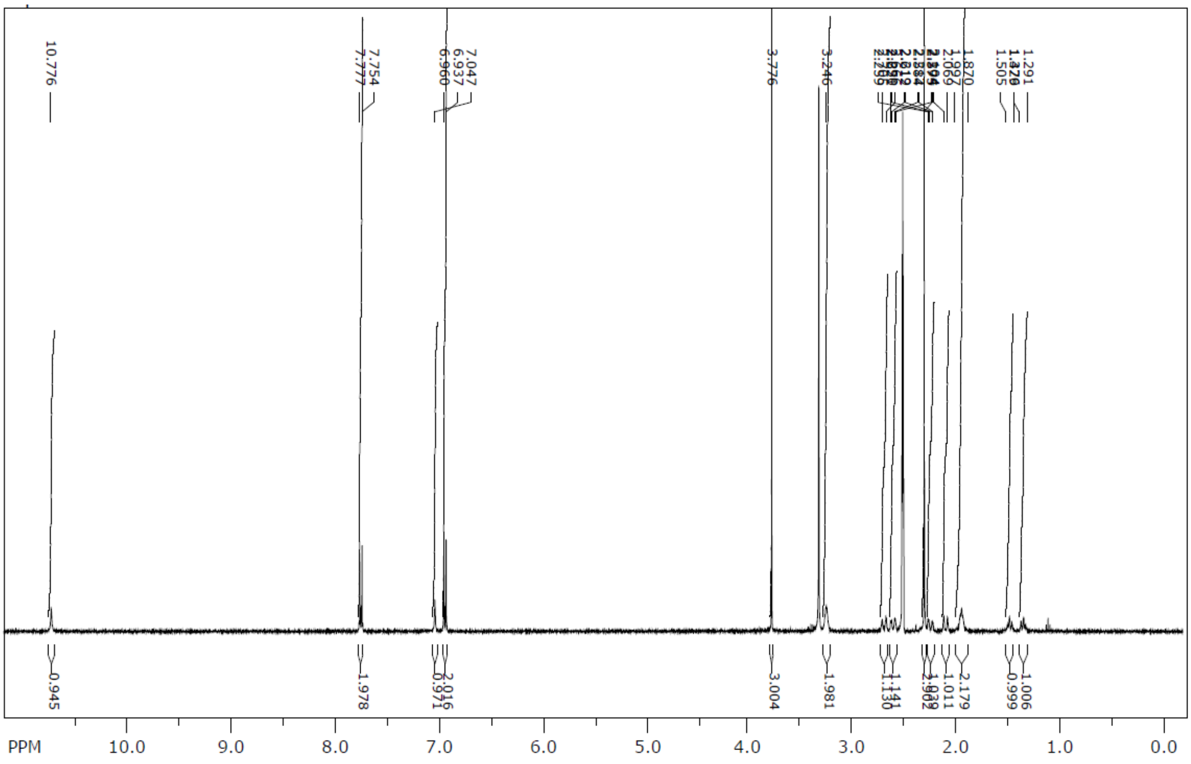

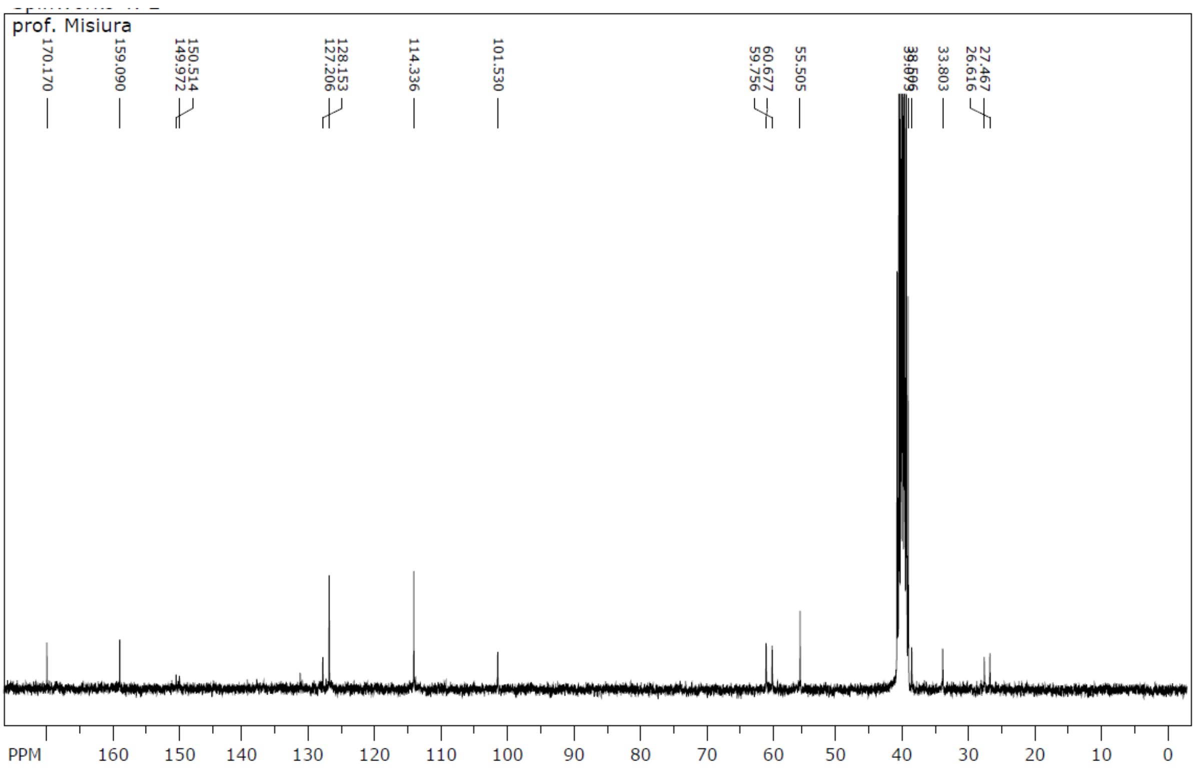

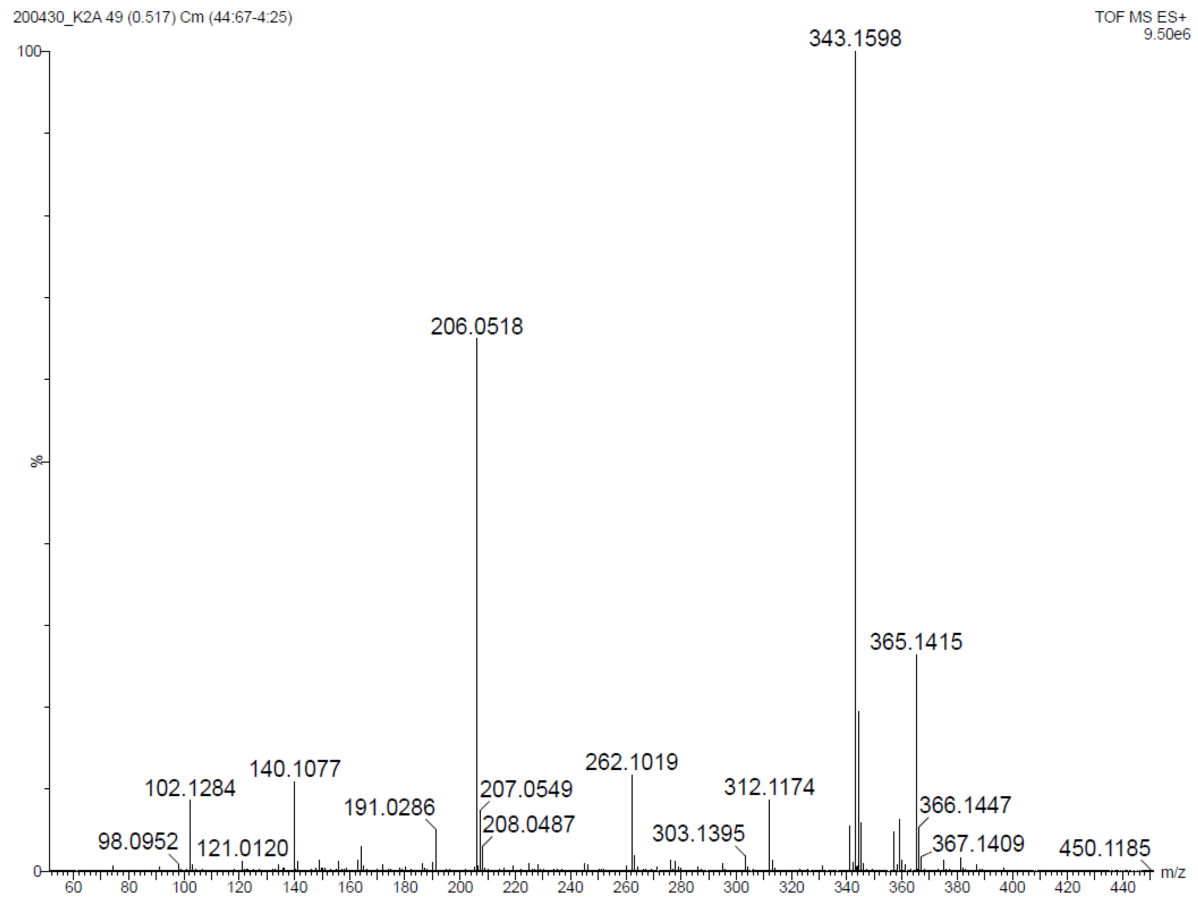
200430_K2A 49 (0.517) Cm (44:67-4:25)
TOF MS ES+
9.50e6
100
%
0
343.1598
206.0518
365.1415
140.1077
262.1019
102.1284
312.1174
191.0286
207.0549
366.1447
208.0487
98.0952
121.0120
303.1395
367.1409
450.1185
m/z
60
80
100
120
140
160
180
200
220
240
260
280
300
320
340
360
380
400
420
440

3c

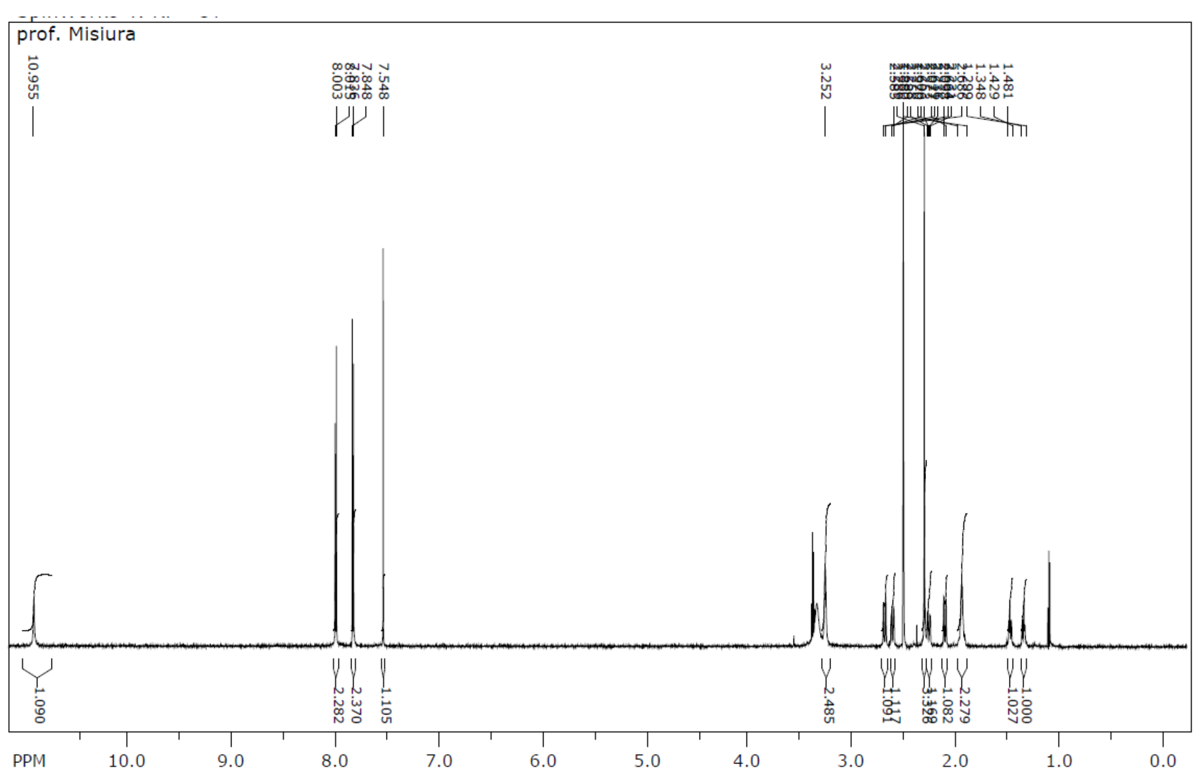
prof. Misiura
10.955
8.003
7.848
7.548
3.252
1.481
1.429
1.348
1.090
2.282
2.370
1.105
2.485
1.091
1.117
1.082
2.279
1.027
1.000
PPM
10.0
9.0
8.0
7.0
6.0
5.0
4.0
3.0
2.0
1.0
0.0

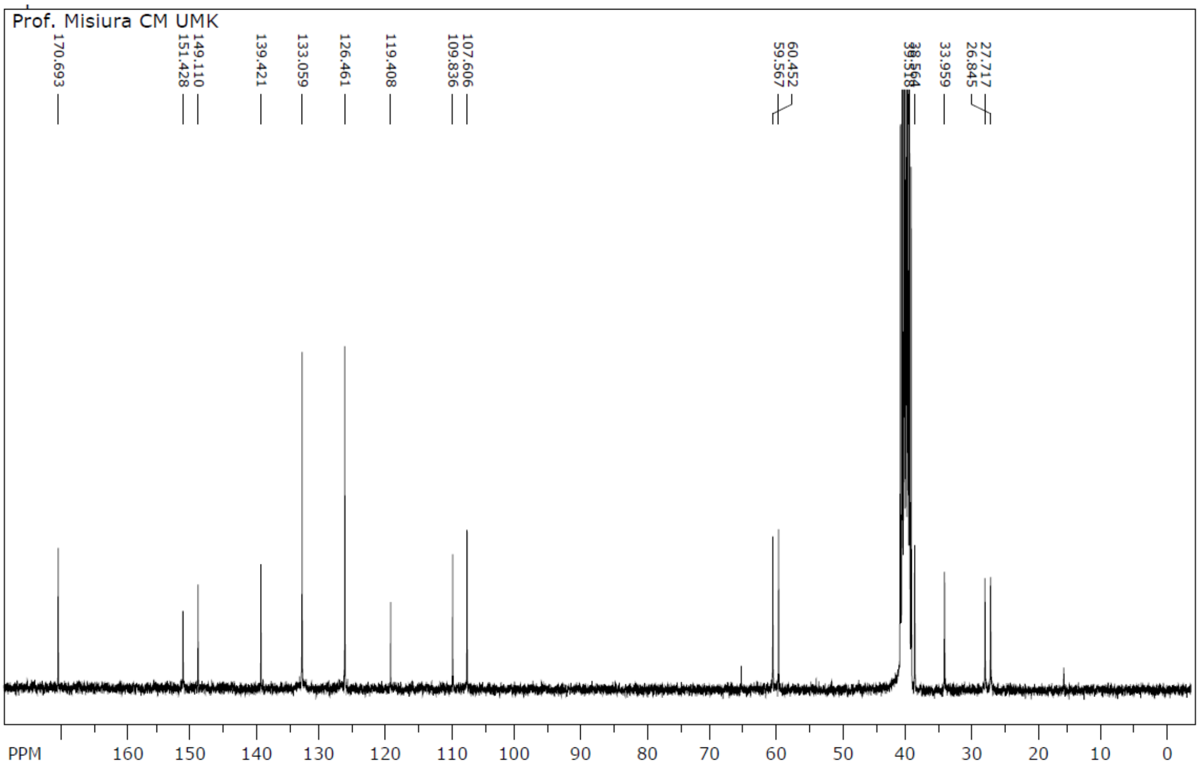

Prof. Misiura CM UMK
170.693
151.428
149.110
139.421
133.059
126.461
119.408
109.836
107.606
60.452
59.567
33.959
27.717
26.845
PPM
160 150 140 130 120 110 100 90 80 70 60 50 40 30 20 10 0

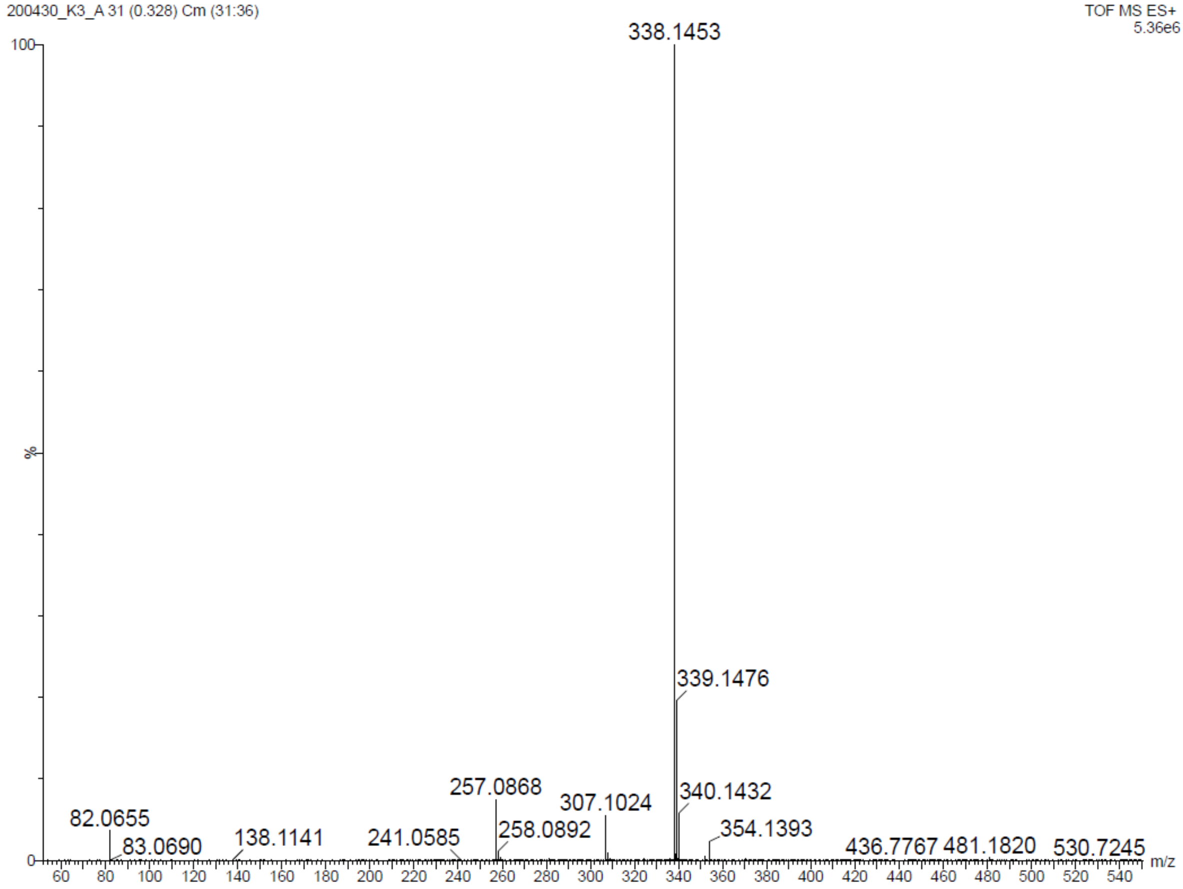

200430_K3_A 31 (0.328) Cm (31:36)
TOF MS ES+
5.36e6
338.1453
339.1476
340.1432
354.1393
307.1024
257.0868
258.0892
241.0585
138.1141
82.0655
83.0690
436.7767
481.1820
530.7245
100
%
0
m/z
60 80 100 120 140 160 180 200 220 240 260 280 300 320 340 360 380 400 420 440 460 480 500 520 540

3d

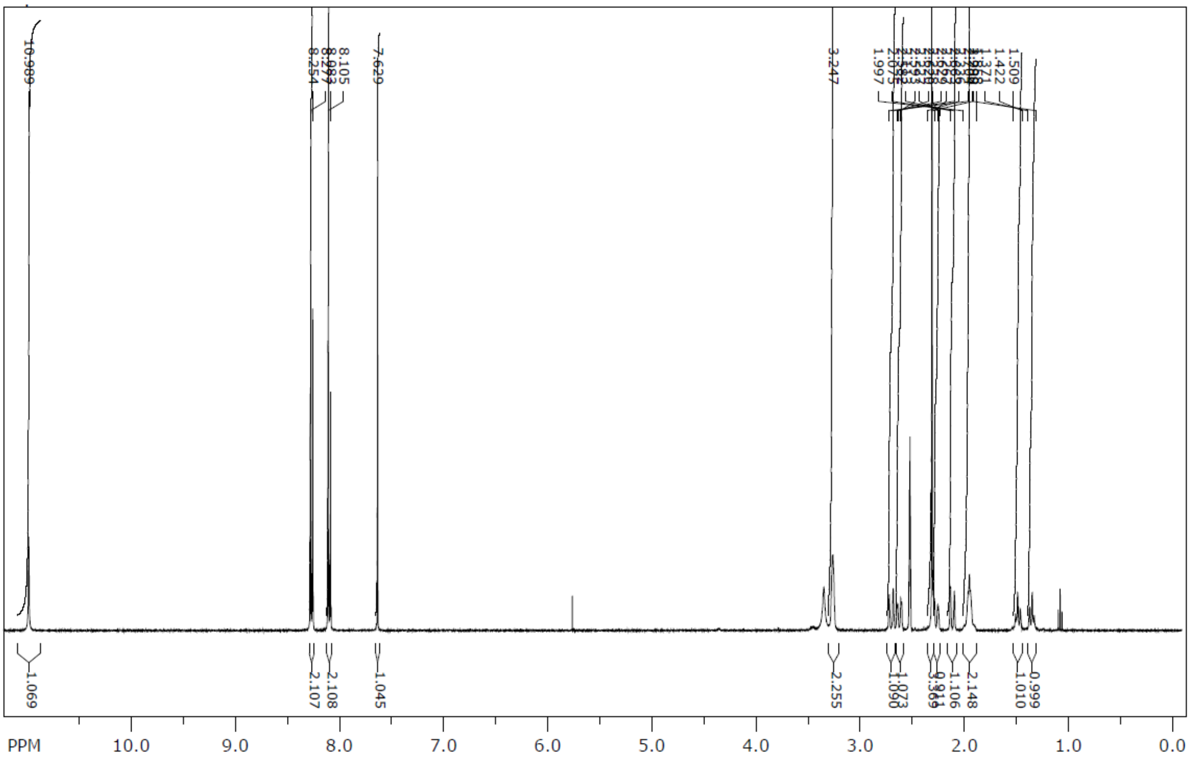

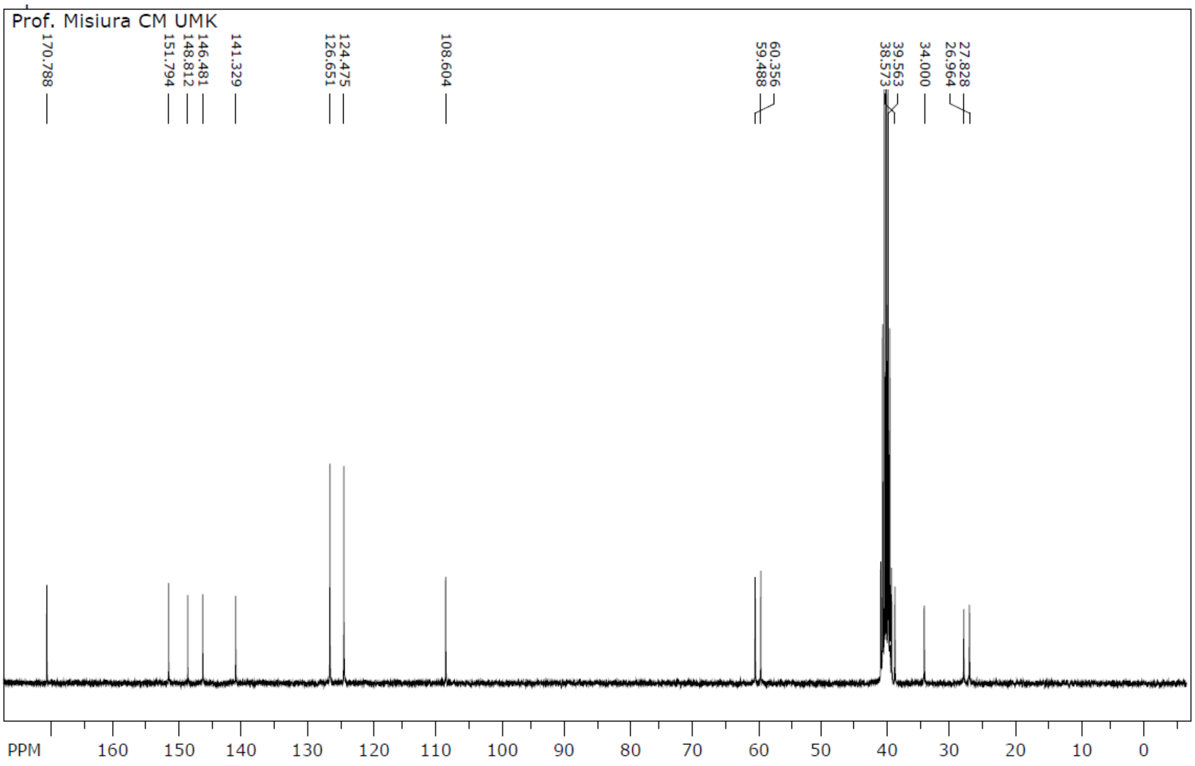
Prof. Misiura CM UMK
170.788
151.794
148.812
146.481
141.329
126.651
124.475
108.604
60.356
59.488
39.563
38.573
34.000
27.828
26.964
PPM
160
150
140
130
120
110
100
90
80
70
60
50
40
30
20
10
0

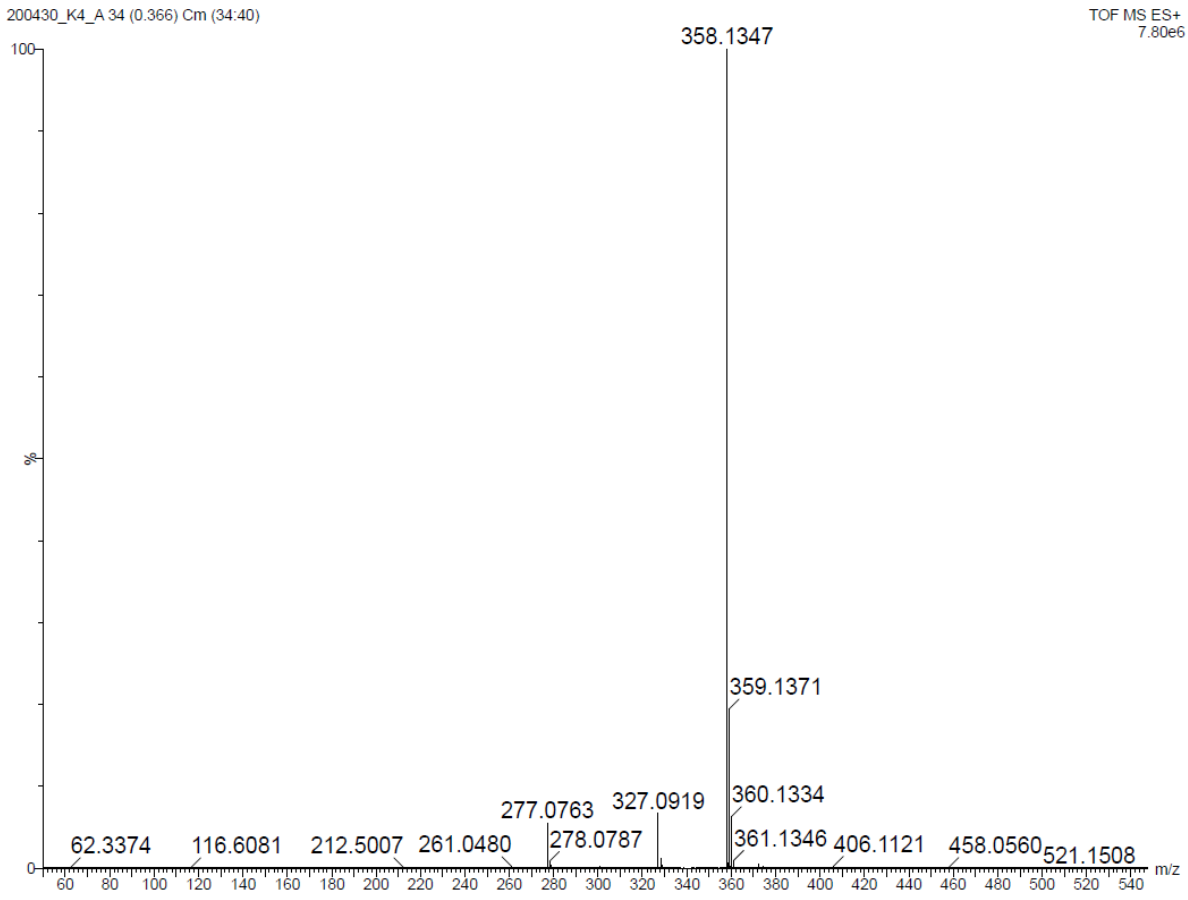
200430_K4_A 34 (0.366) Cm (34:40)
TOF MS ES+
7.80e6
358.1347
359.1371
360.1334
361.1346
327.0919
277.0763
278.0787
62.3374
116.6081
212.5007
261.0480
406.1121
458.0560
521.1508
%
m/z

3e

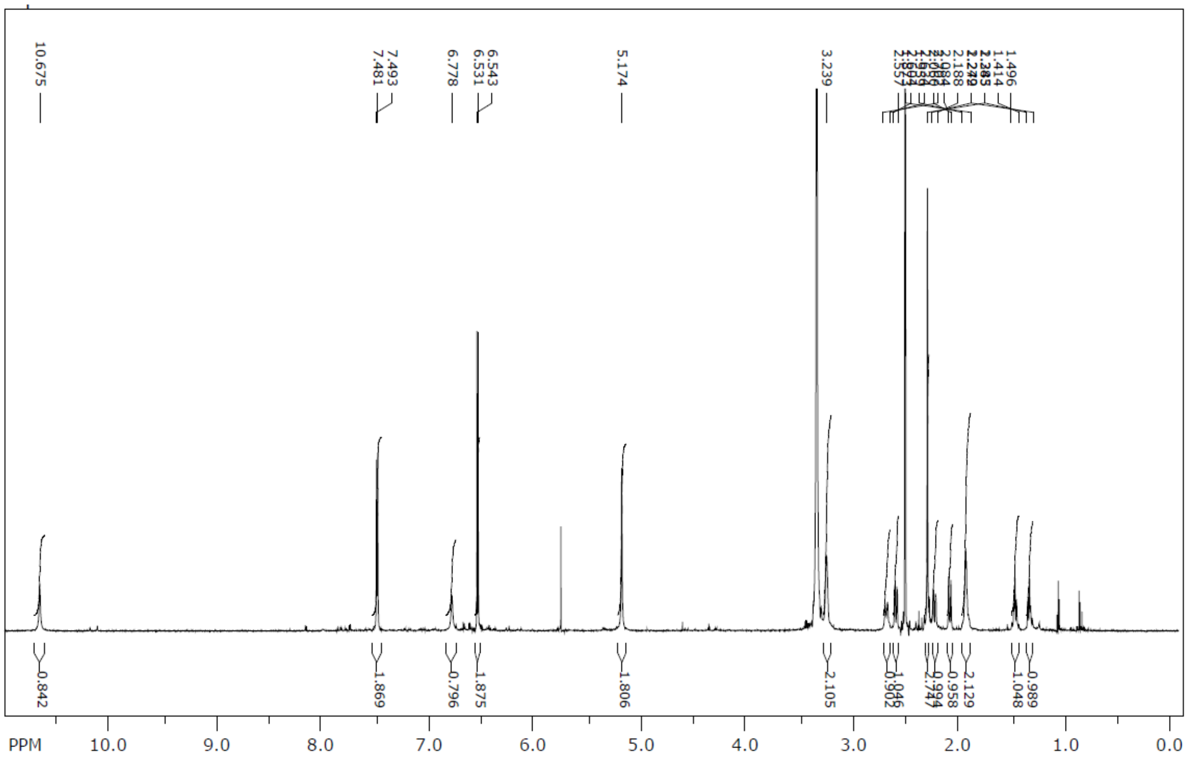
10.675
7.493
7.481
6.778
6.543
6.531
5.174
3.239
1.496
1.414
0.842
1.869
0.796
1.875
1.806
2.105
2.129
1.048
0.989
0.958
PPM

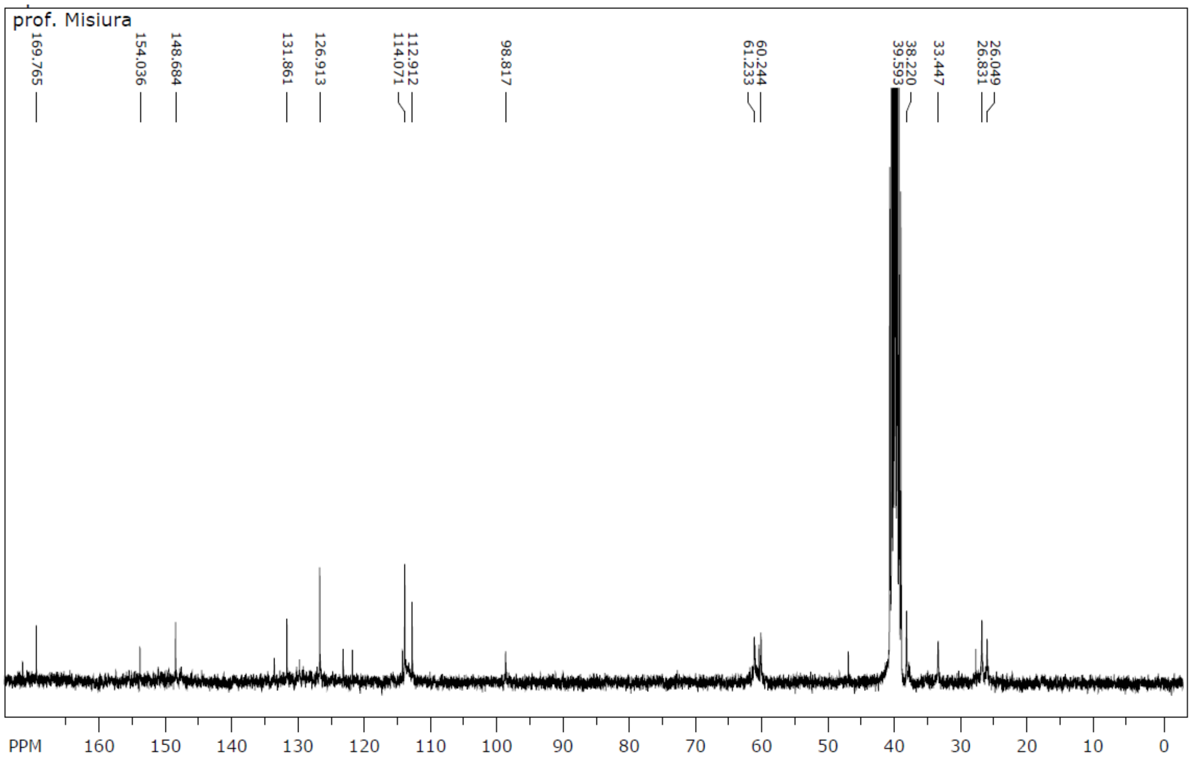

prof. Misiura
169.765
154.036
148.684
131.861
126.913
114.071
112.912
98.817
61.233
60.244
39.593
38.220
33.447
26.831
26.049
PPM
160
150
140
130
120
110
100
90
80
70
60
50
40
30
20
10
0

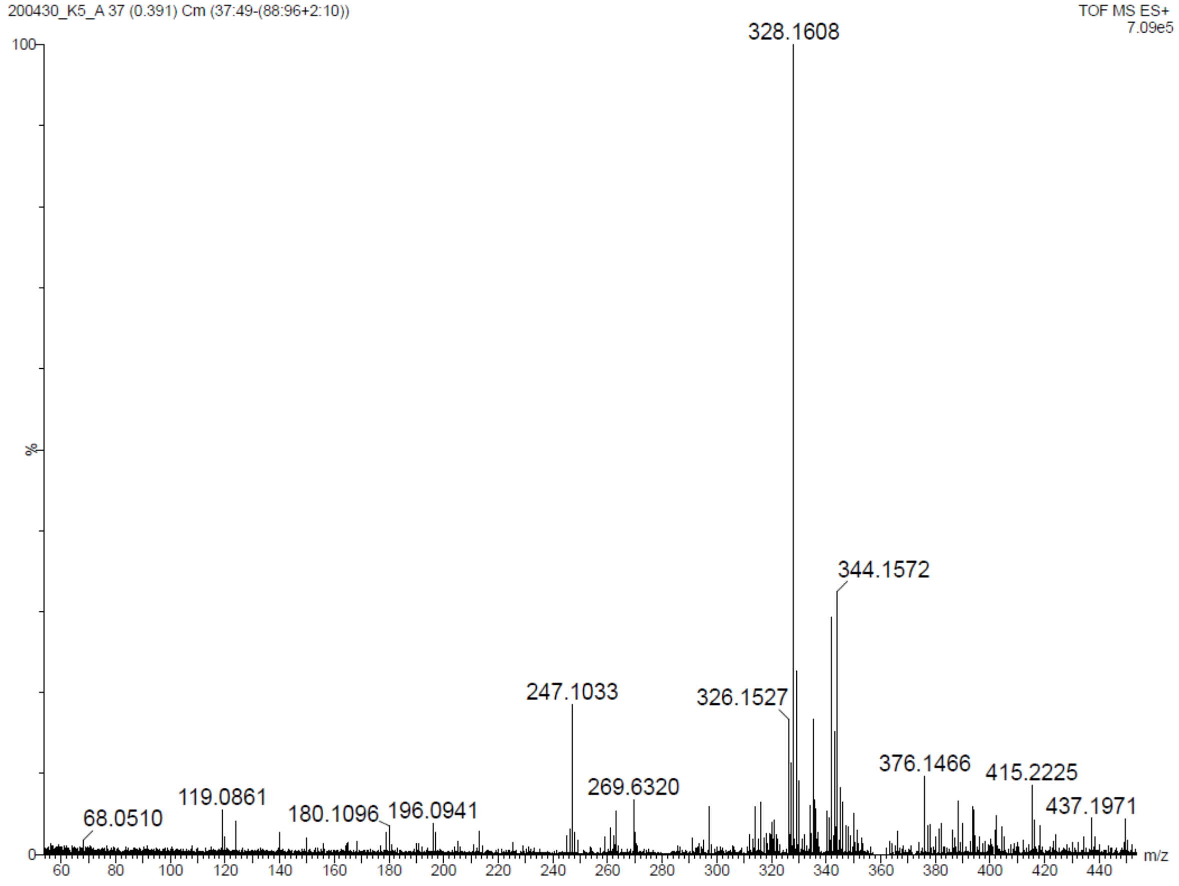

200430_K5_A 37 (0.391) Cm (37:49-(88:96+2:10))
TOF MS ES+
7.09e5
328.1608
344.1572
326.1527
247.1033
376.1466
415.2225
437.1971
269.6320
119.0861
180.1096
196.0941
68.0510
100
%
0
m/z

3f

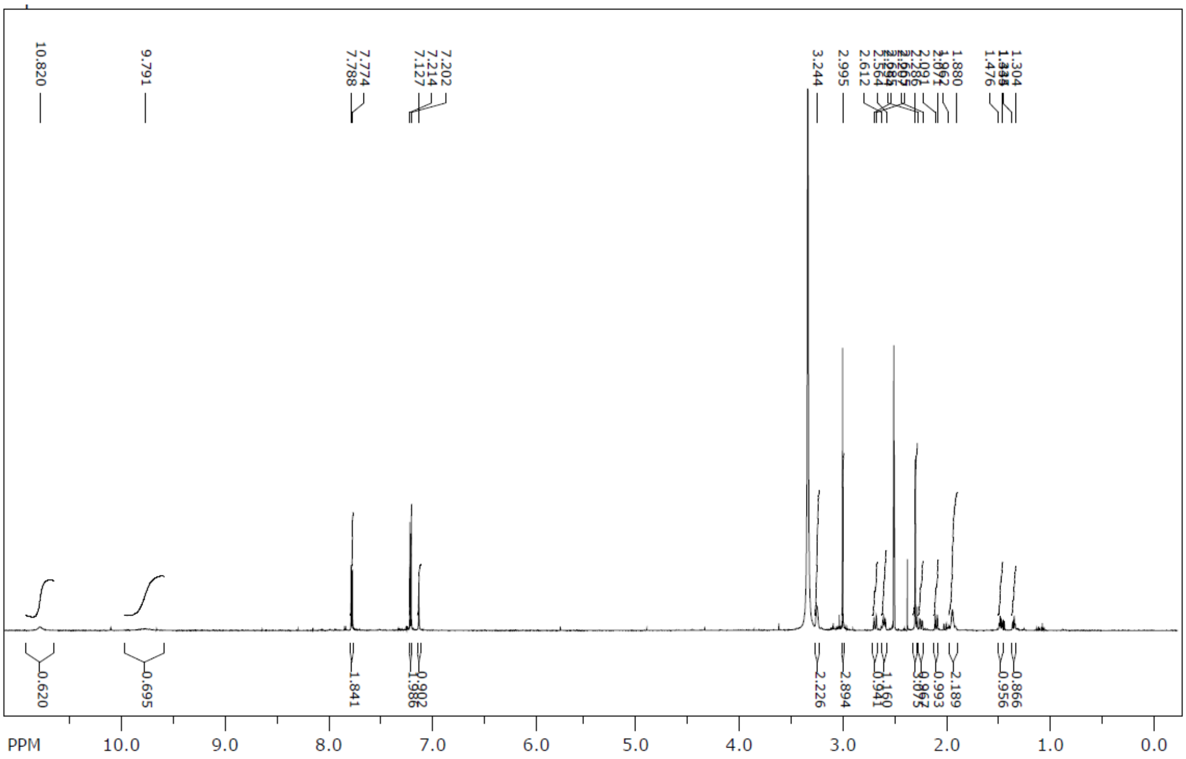
10.820
9.791
7.774
7.788
7.202
7.214
7.127
3.244
2.995
2.612
2.564
2.091
2.071
1.962
1.880
1.476
1.304
0.620
0.695
1.841
0.902
2.226
2.894
0.941
1.160
0.993
2.189
0.956
0.866
PPM
10.0
9.0
8.0
7.0
6.0
5.0
4.0
3.0
2.0
1.0
0.0

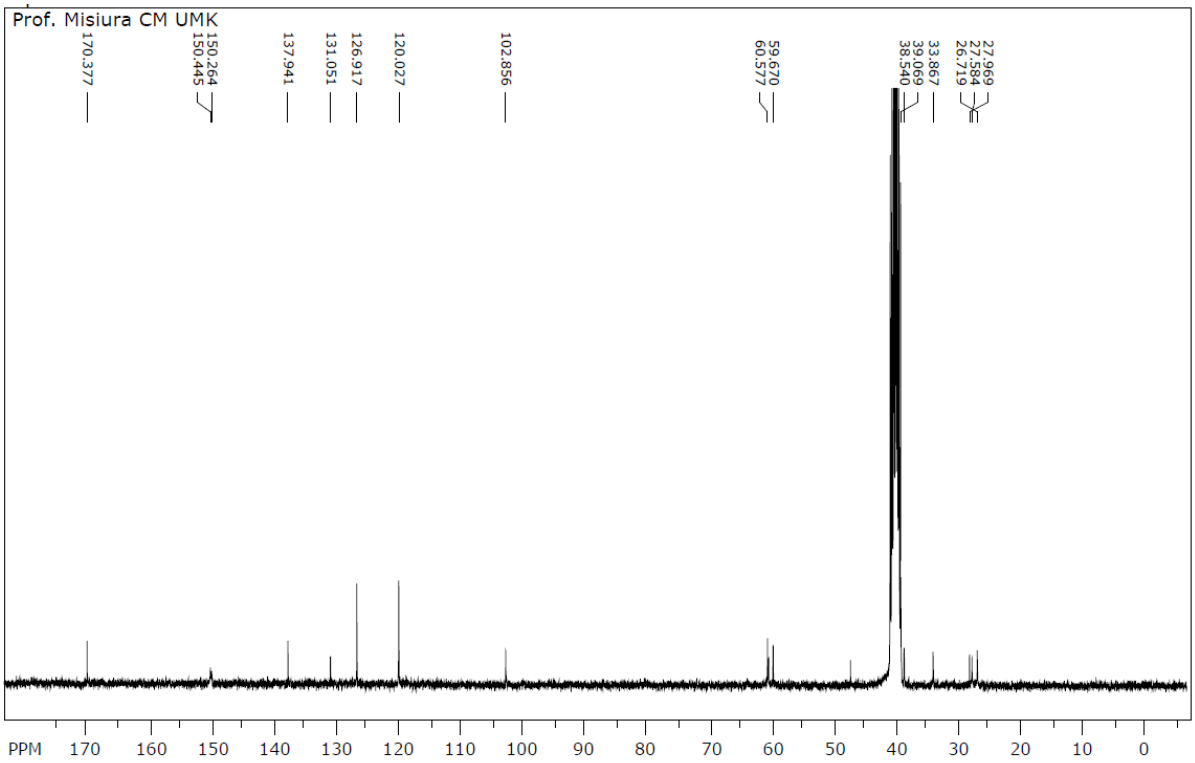
Prof. Misiura CM UMK
170.377
150.264
150.445
137.941
131.051
126.917
120.027
102.856
59.670
60.577
39.069
38.540
33.867
27.969
27.584
26.719
PPM
170
160
150
140
130
120
110
100
90
80
70
60
50
40
30
20
10
0

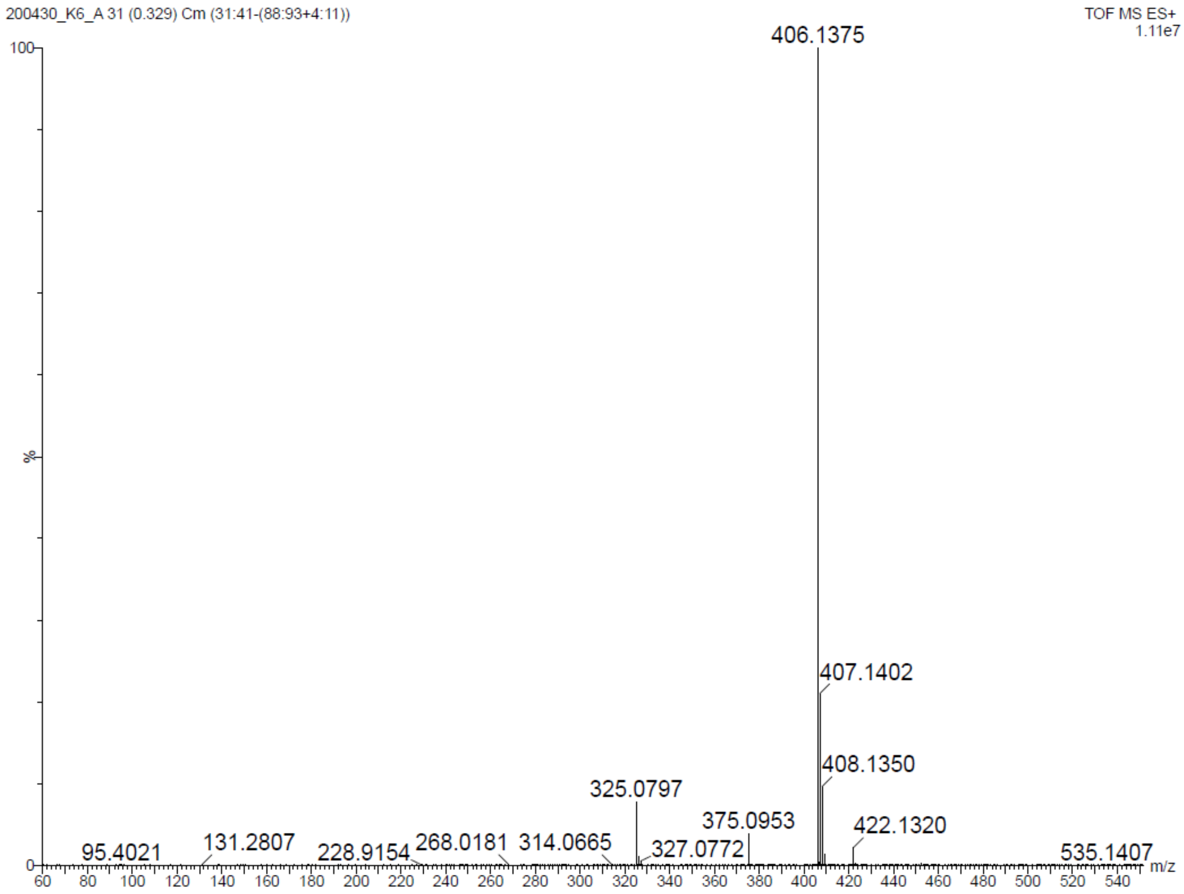

200430_K6_A 31 (0.329) Cm (31:41-(88:93+4:11))
TOF MS ES+
1.11e7
406.1375
407.1402
408.1350
325.0797
375.0953
422.1320
95.4021
131.2807
228.9154
268.0181
314.0665
327.0772
535.1407
m/z
%

3g

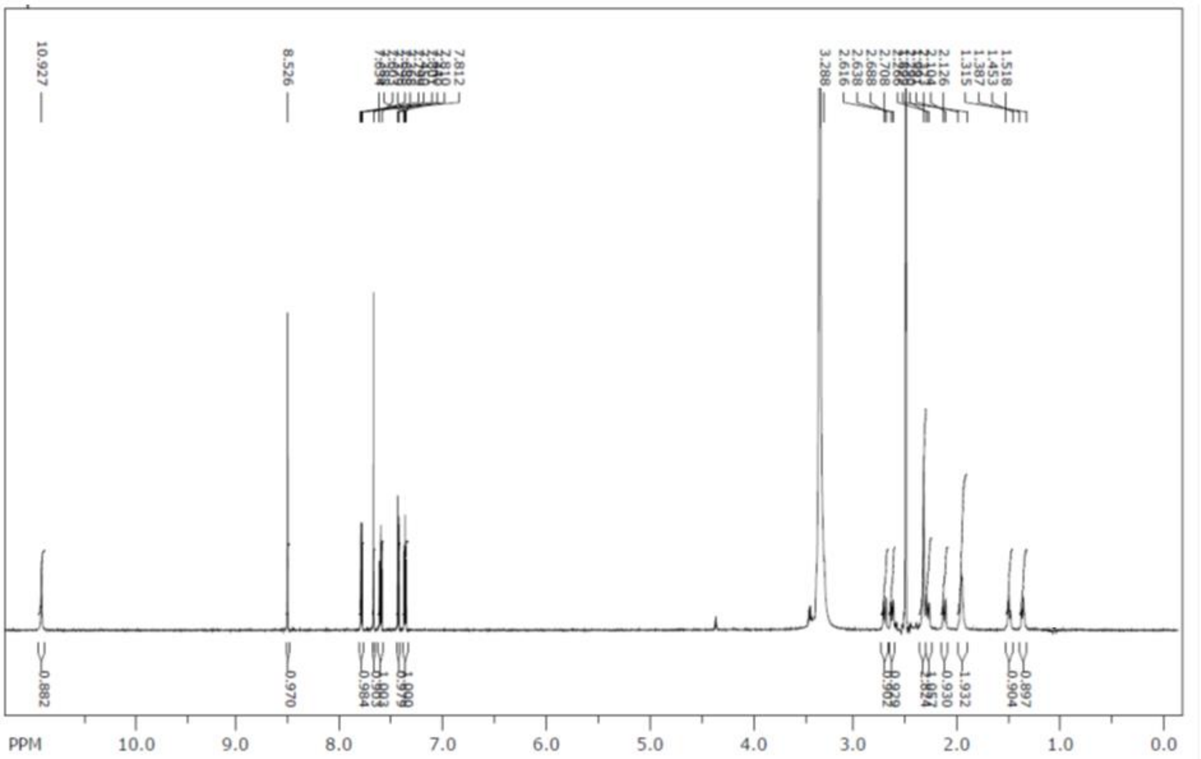

10.927
8.526
7.812
3.288
2.616
2.638
2.688
2.708
2.126
2.104
1.518
1.453
1.387
1.315
0.882
0.970
0.984
1.932
0.930
0.904
0.897
PPM

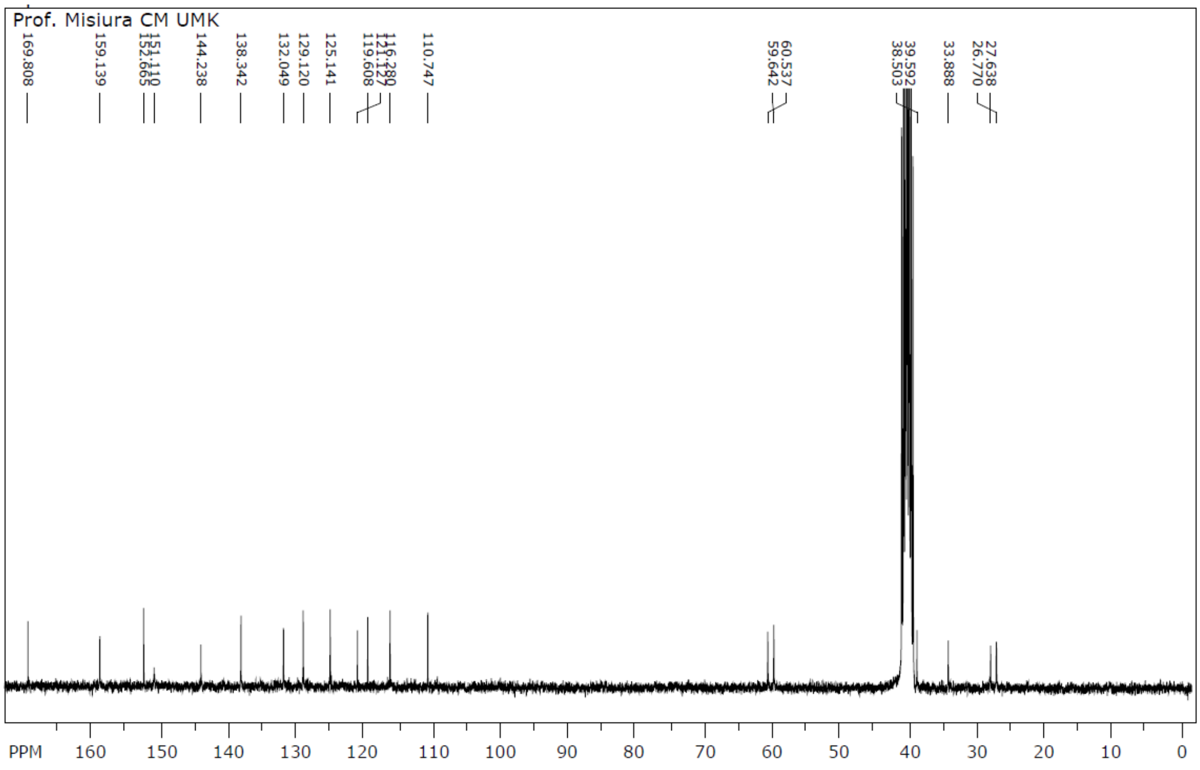
Prof. Misiura CM UMK
169.808
159.139
152.665
151.110
144.238
138.342
132.049
129.120
125.141
119.608
116.280
110.747
60.537
59.642
39.592
38.503
33.888
27.638
26.770
PPM

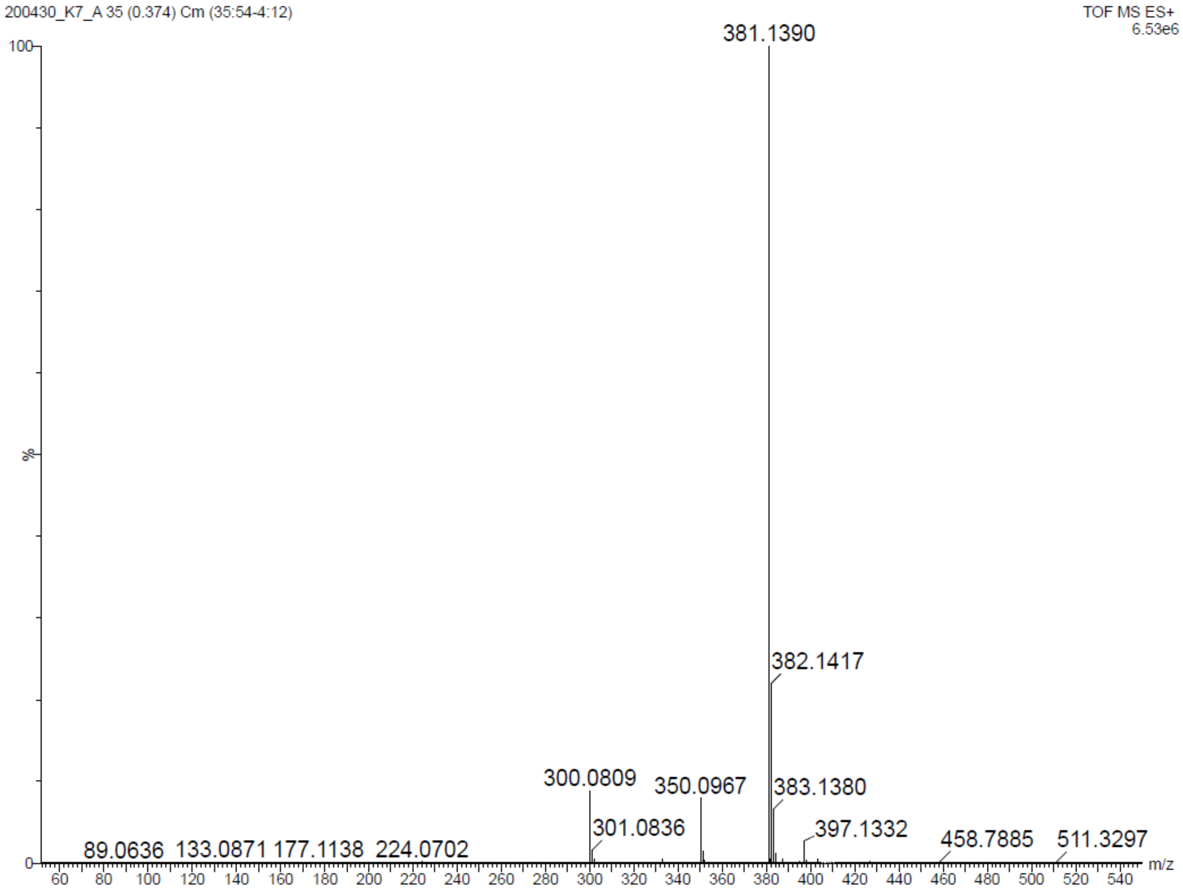
200430_K7_A 35 (0.374) Cm (35:54-4:12)
TOF MS ES+
6.53e6
381.1390
382.1417
300.0809
350.0967
383.1380
301.0836
397.1332
458.7885
511.3297
89.0636
133.0871
177.1138
224.0702
%
m/z

3h

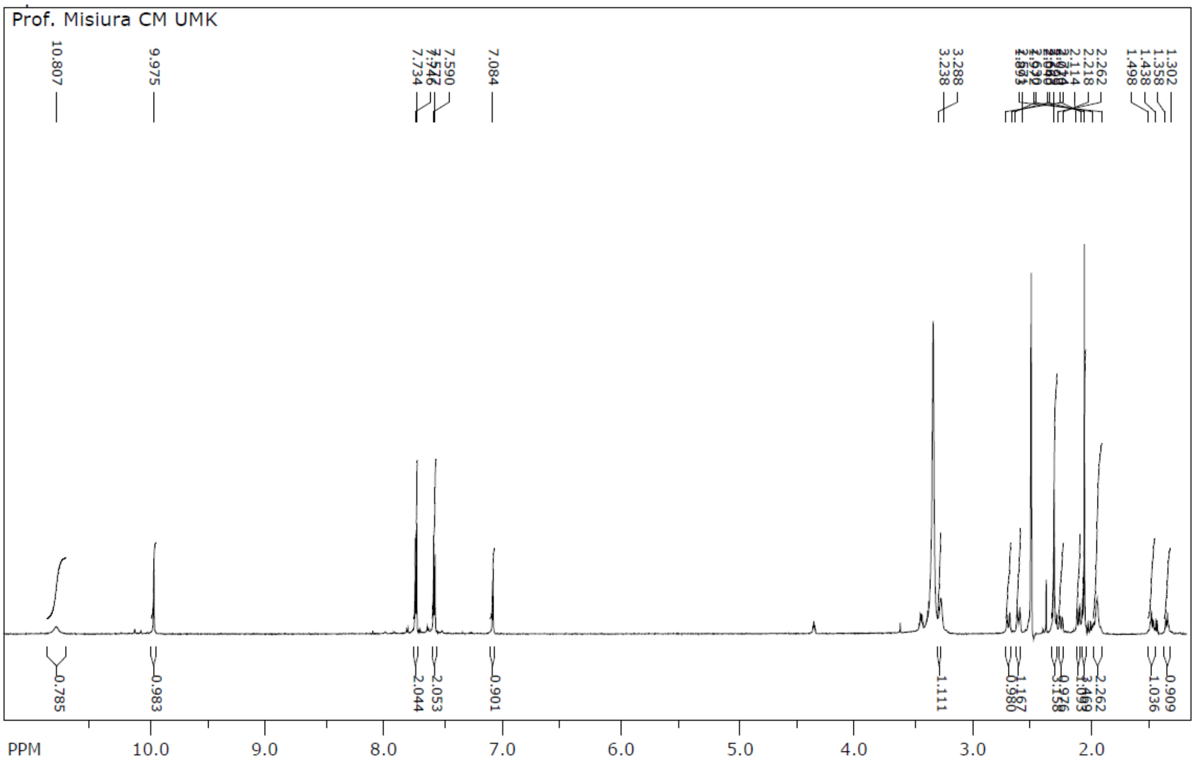
Prof. Misiura CM UMK
10.807
9.975
7.590
7.734
7.084
3.288
3.238
2.262
2.218
2.114
1.498
1.438
1.358
1.302
0.785
0.983
2.044
2.053
0.901
1.111
0.980
1.167
0.976
2.262
1.036
0.909
PPM
10.0
9.0
8.0
7.0
6.0
5.0
4.0
3.0
2.0

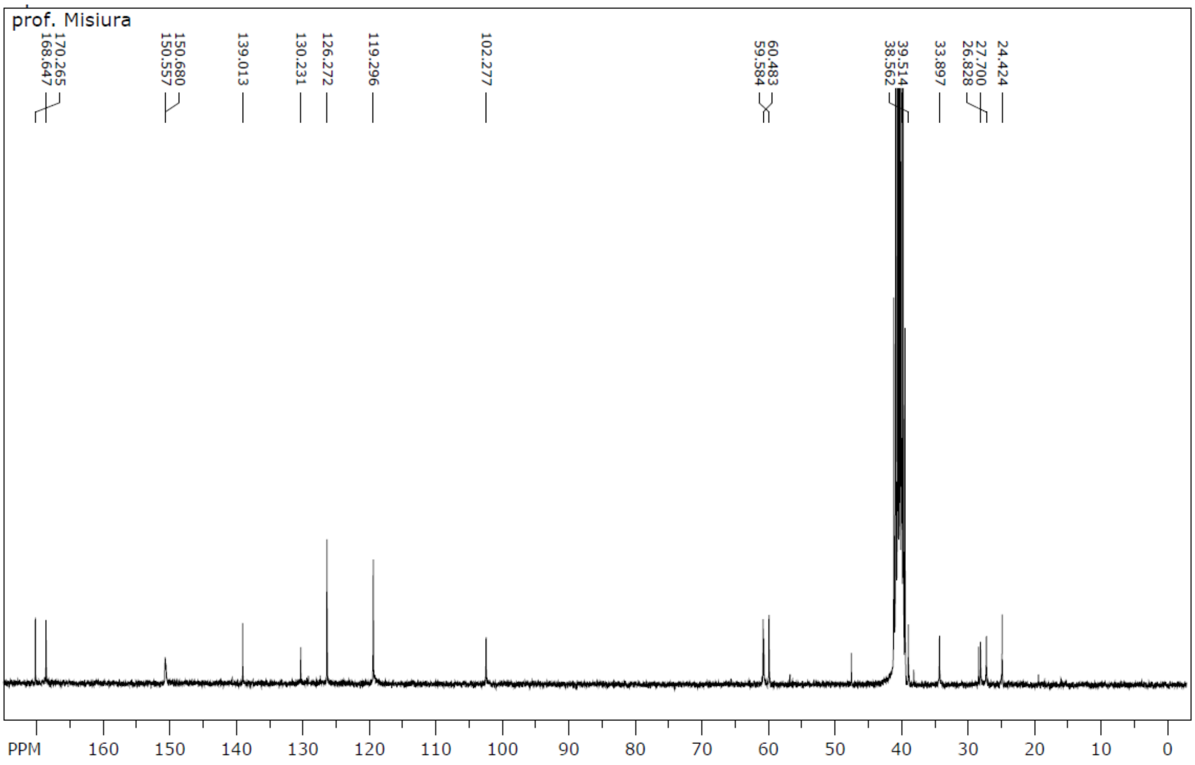
prof. Misiura
170.265
168.647
150.680
150.557
139.013
130.231
126.272
119.296
102.277
60.483
59.584
39.514
38.562
33.897
27.700
26.828
24.424
PPM
160
150
140
130
120
110
100
90
80
70
60
50
40
30
20
10
0

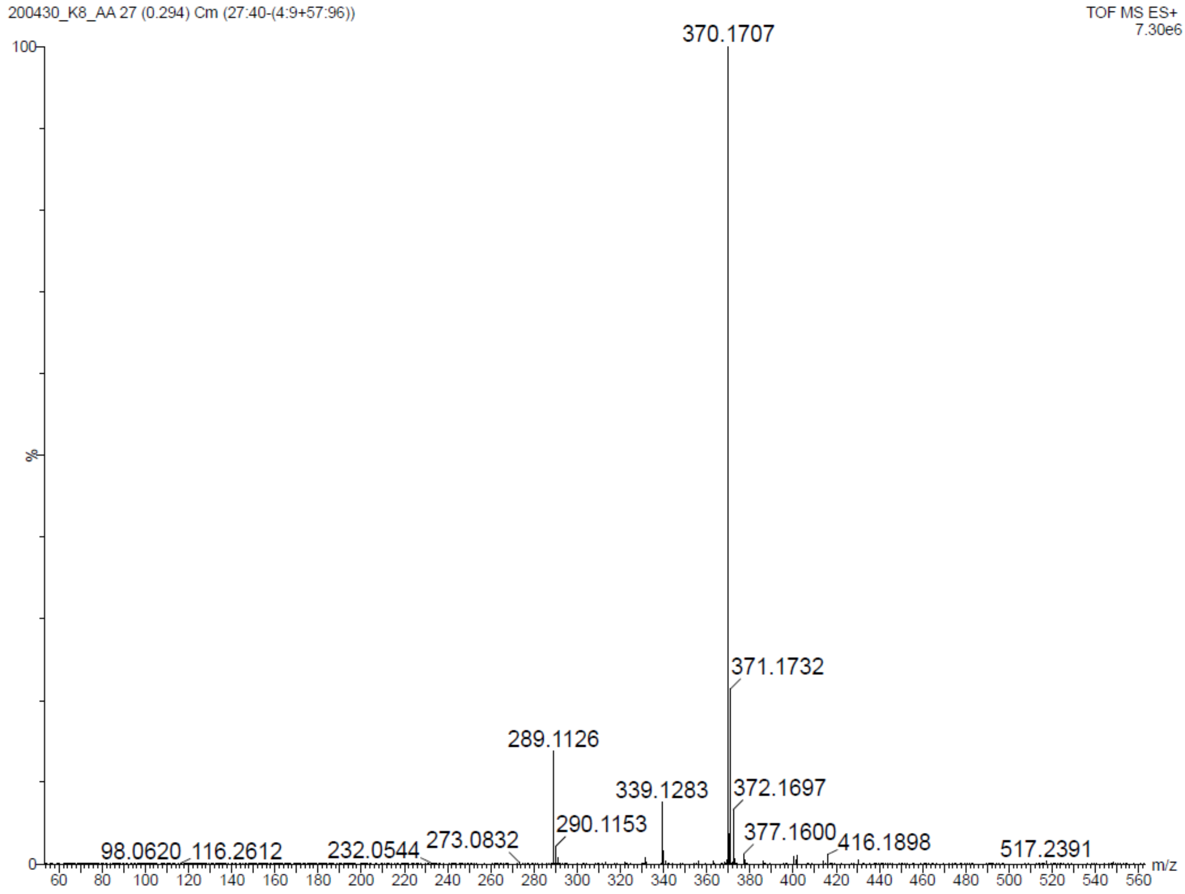
200430_K8_AA 27 (0.294) Cm (27:40-(4:9+57:96))
TOF MS ES+
7.30e6
370.1707
100
%
0
371.1732
289.1126
339.1283
372.1697
290.1153
377.1600
416.1898
98.0620
116.2612
232.0544
273.0832
517.2391
60
80
100
120
140
160
180
200
220
240
260
280
300
320
340
360
380
400
420
440
460
480
500
520
540
560
m/z

3a-HOMO

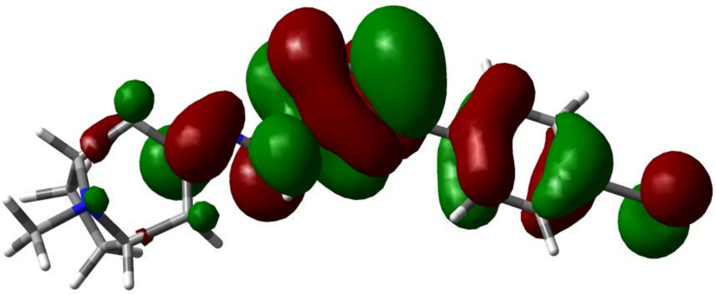

3a-LUMO

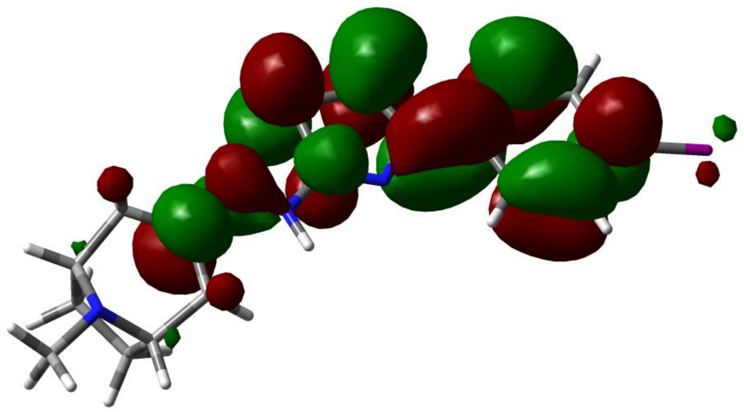

3b-HOMO

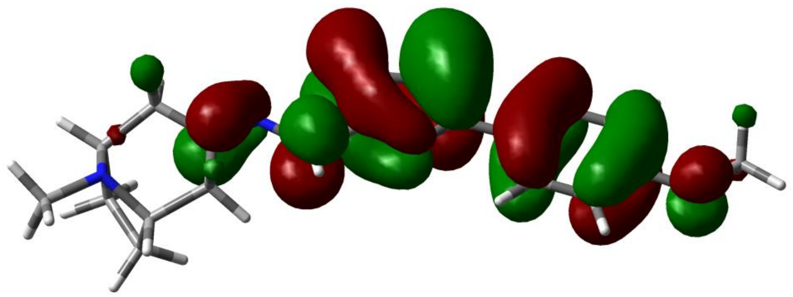

3b-LUMO

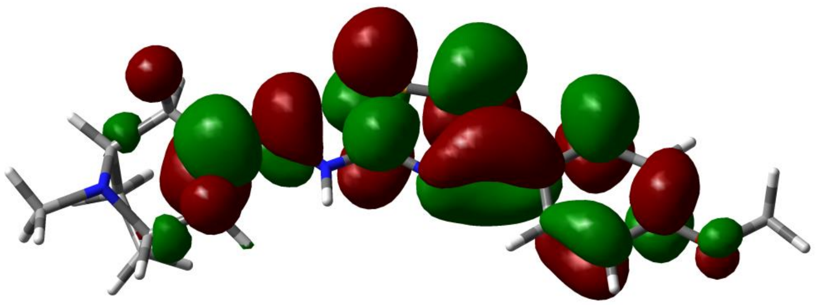

3c-HOMO

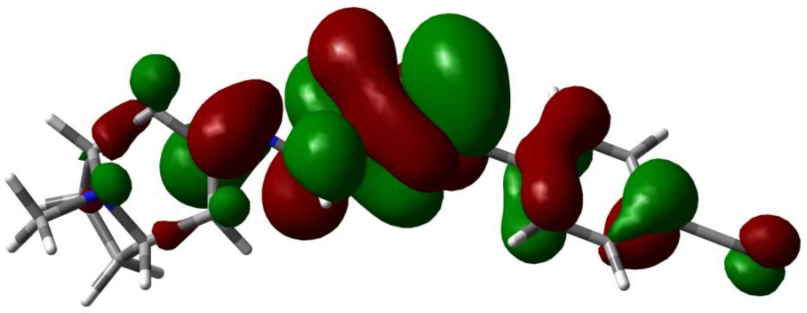

3c-LUMO

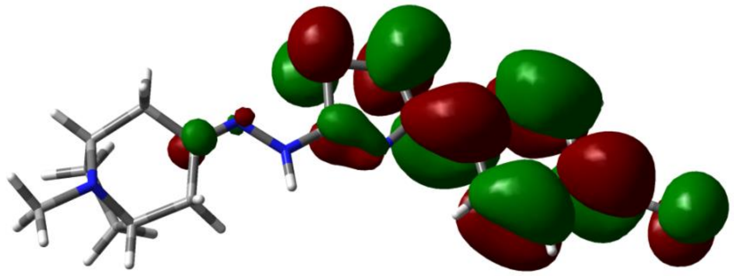

3d-HOMO

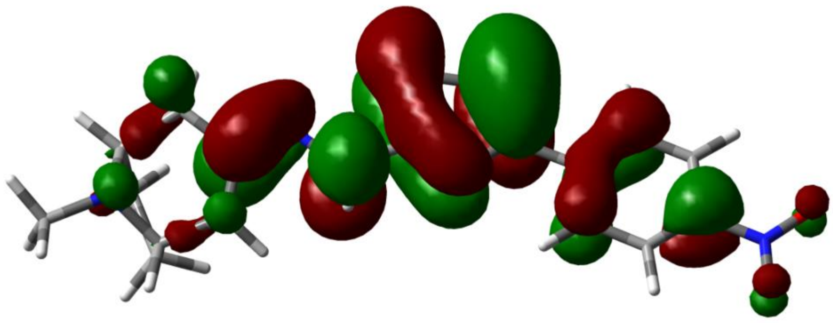

3d-LUMO

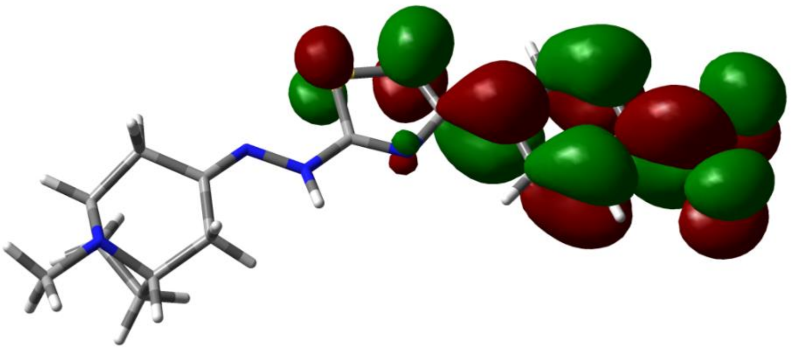

3e-HOMO

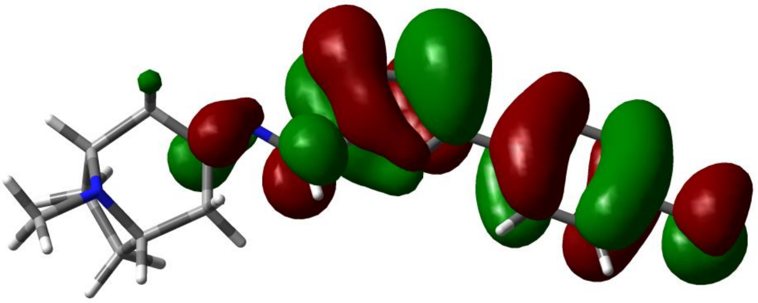

3e-LUMO

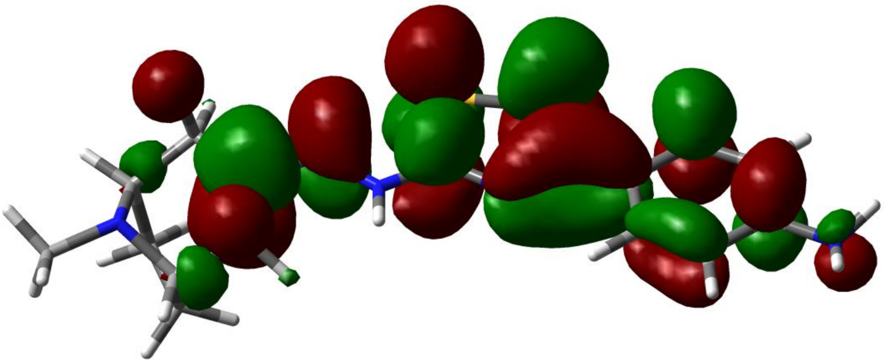

3f-HOMO

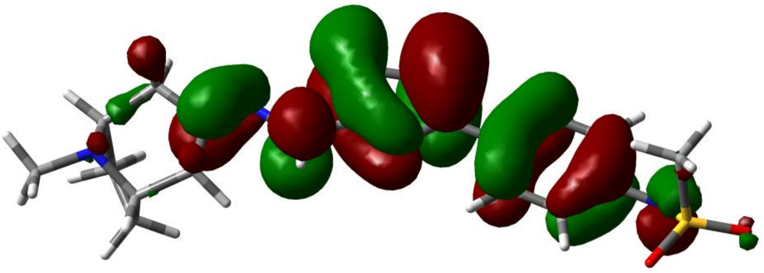

3f-LUMO

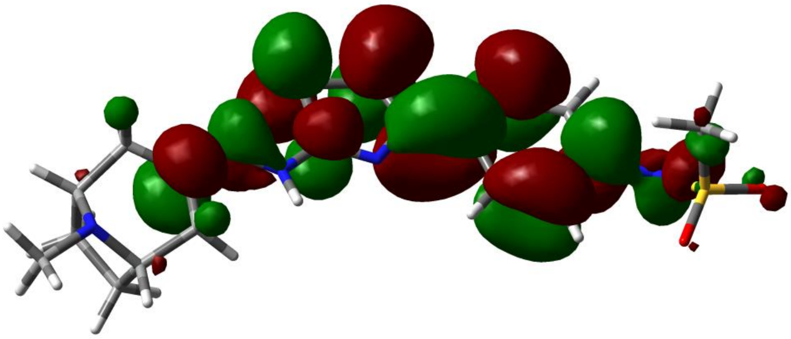

3g-HOMO

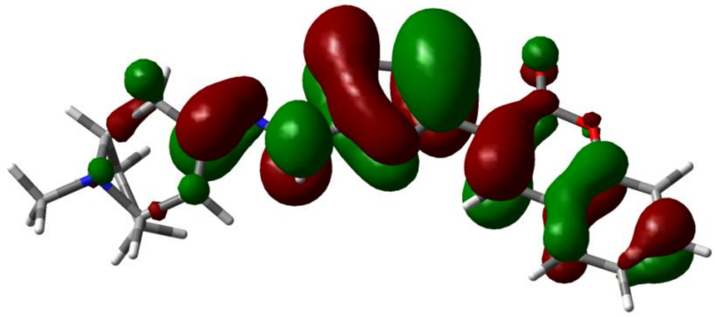

3g-LUMO

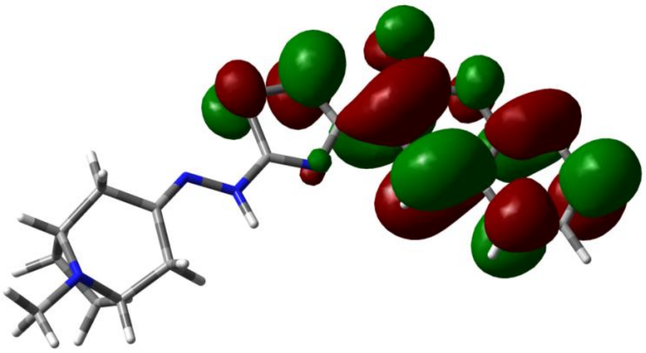

3h-HOMO

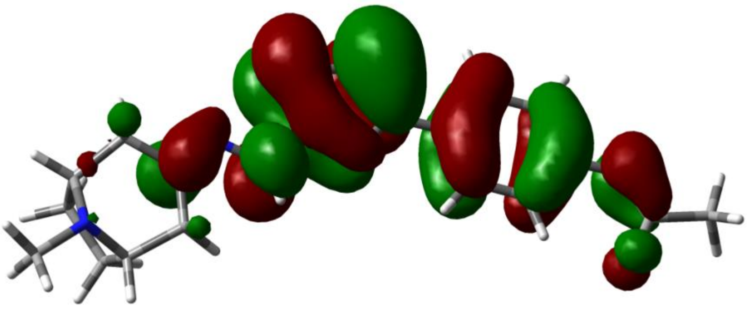

3h-LUMO

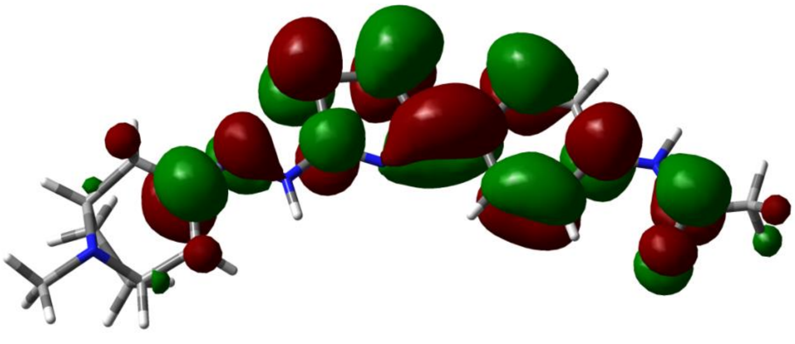

Geometrical parameters of the investigated compounds **3a**-**3h**.

3a

C	6.219519	-1.800700	0.071662
C	6.281359	-1.356748	1.564416
C	6.954401	0.041600	1.504665
C	7.206689	0.264051	-0.015559
N	7.318579	-1.087679	-0.602102
C	6.013896	0.954744	-0.699862
C	4.895781	-1.363732	-0.592391
C	4.747389	0.143257	-0.548161
C	8.626767	-1.708985	-0.408964
N	3.651911	0.793698	-0.399481
N	2.470416	0.125229	-0.312440
C	1.305809	0.832471	-0.175670
S	1.318609	2.576170	0.049455
C	-0.422897	2.500634	0.132989
C	-0.866141	1.216848	-0.013024
N	0.135730	0.274015	-0.187837
C	-2.268831	0.764373	-0.000011
C	-2.565270	-0.596588	-0.153636
C	-3.879369	-1.054913	-0.145404
C	-4.917732	-0.145308	0.018293
C	-4.654770	1.213195	0.172954
C	-3.337830	1.657268	0.162632
I	-6.942665	-0.836968	0.032705
H	6.341169	-2.878785	-0.051467
H	6.881517	-2.058191	2.147476

H	5.290735	-1.325979	2.023239
H	6.325931	0.826668	1.929528
H	7.895876	0.048034	2.057680
H	8.117932	0.832485	-0.212343
H	6.251067	1.046747	-1.766349
H	5.843454	1.958804	-0.305730
H	4.902810	-1.702454	-1.635231
H	4.062227	-1.859996	-0.085118
H	8.622191	-2.705102	-0.858070
H	8.937044	-1.811773	0.644372
H	9.384426	-1.116507	-0.927115
H	2.374656	-0.857629	-0.537940
H	-0.989344	3.405358	0.278662
H	-1.748365	-1.294672	-0.280313
H	-4.084366	-2.110791	-0.265802
H	-5.463604	1.920860	0.299928
H	-3.150942	2.717851	0.283426

3b

C	4.991037	-1.597393	0.058782
C	5.018879	-1.158218	1.554001
C	5.571930	0.291954	1.501210
C	5.801135	0.543627	-0.018144
N	6.025120	-0.790715	-0.612642
C	4.552908	1.135300	-0.696305
C	3.633701	-1.269475	-0.599762
C	3.359217	0.219583	-0.546300

C	7.381037	-1.300949	-0.424712
N	2.213139	0.774373	-0.390638
N	1.093145	0.009590	-0.303496
C	-0.130029	0.614099	-0.164904
S	-0.272727	2.349491	0.067185
C	-2.002642	2.117432	0.147418
C	-2.330283	0.800109	-0.006882
N	-1.244736	-0.046875	-0.182240
C	-3.685094	0.220229	0.002408
C	-3.856836	-1.172228	-0.079335
C	-5.118190	-1.742862	-0.067724
C	-6.258721	-0.937564	0.025422
C	-6.109987	0.450300	0.104273
C	-4.835327	1.010678	0.090837
O	-7.455344	-1.593180	0.029581
C	-8.646369	-0.826472	0.122247
H	5.203000	-2.660733	-0.070673
H	5.676872	-1.810113	2.132255
H	4.029911	-1.212892	2.014090
H	4.880488	1.018850	1.931674
H	6.510883	0.374319	2.052583
H	6.661196	1.187580	-0.213068
H	4.779668	1.253290	-1.762569
H	4.299732	2.119225	-0.295828
H	3.665803	-1.601779	-1.644265
H	2.846123	-1.836181	-0.092830
H	7.459571	-2.291195	-0.880057

H	7.700898	-1.384025	0.627617
H	8.085502	-0.643682	-0.939888
H	1.078963	-0.975080	-0.540721
H	-2.647040	2.966255	0.304937
H	-2.977063	-1.797790	-0.152446
H	-5.249403	-2.816556	-0.129838
H	-6.971372	1.101018	0.172760
H	-4.749696	2.089864	0.145839
H	-8.684484	-0.252015	1.054873
H	-8.753402	-0.143464	-0.728216
H	-9.464641	-1.544817	0.110083

3c

C	4.799742	-1.615901	0.069242
C	4.826016	-1.171228	1.562742
C	5.383521	0.277154	1.505360
C	5.615770	0.522235	-0.014509
N	5.836256	-0.814571	-0.603943
C	4.370584	1.115260	-0.697292
C	3.444524	-1.286896	-0.593727
C	3.174302	0.202981	-0.548391
C	7.191050	-1.328245	-0.414776
N	2.029434	0.762396	-0.401886
N	0.905437	-0.000925	-0.318147
C	-0.310214	0.609762	-0.178520
S	-0.432981	2.352064	0.037354
C	-2.160906	2.142373	0.128516

C	-2.503539	0.826384	-0.008650
N	-1.433752	-0.037837	-0.182523
C	-3.866043	0.269947	0.014301
C	-4.999684	1.084030	0.172649
C	-6.273235	0.540951	0.192676
C	-6.450648	-0.844542	0.053022
C	-5.326671	-1.666823	-0.106483
C	-4.055353	-1.113862	-0.125118
C	-7.763394	-1.408707	0.073328
N	-8.825444	-1.864466	0.090013
H	5.008265	-2.680277	-0.056116
H	5.481200	-1.822797	2.144303
H	3.836386	-1.221784	2.022042
H	4.694368	1.008060	1.932799
H	6.321993	0.358466	2.057454
H	6.477474	1.163342	-0.210744
H	4.599014	1.228181	-1.763613
H	4.118955	2.101329	-0.301263
H	3.478763	-1.621959	-1.637161
H	2.654193	-1.850132	-0.087182
H	7.266482	-2.319959	-0.867197
H	7.510491	-1.408832	0.637686
H	7.897038	-0.674582	-0.932226
H	0.891649	-0.990507	-0.533930
H	-2.795709	3.000782	0.272969
H	-4.889321	2.156003	0.281117
H	-7.138425	1.180638	0.315470

H	-5.458512	-2.736405	-0.214899
H	-3.185053	-1.744012	-0.247981

3d

C	5.181681	-1.660712	0.082789
C	5.212806	-1.208421	1.573915
C	5.803372	0.226583	1.510578
C	6.046035	0.457927	-0.009800
N	6.238265	-0.886739	-0.591133
C	4.816769	1.075062	-0.700043
C	3.836494	-1.304855	-0.586884
C	3.599773	0.190881	-0.550002
C	7.580507	-1.429804	-0.394439
N	2.467573	0.776969	-0.409582
N	1.326094	0.039571	-0.325312
C	0.125288	0.678515	-0.189629
S	0.044070	2.425122	0.016949
C	-1.687481	2.257554	0.108286
C	-2.061821	0.949061	-0.021827
N	-1.013542	0.058222	-0.190716
C	-3.437238	0.427609	0.003204
C	-4.549051	1.272411	0.166080
C	-5.836947	0.763202	0.187617
C	-6.021361	-0.609773	0.044317
C	-4.944571	-1.474443	-0.119057
C	-3.659685	-0.952111	-0.138769
N	-7.388831	-1.156682	0.066604

O	-7.520398	-2.368073	-0.061569
O	-8.315481	-0.367927	0.212174
H	5.366392	-2.730201	-0.036044
H	5.850940	-1.871462	2.161408
H	4.220718	-1.233953	2.029989
H	5.129792	0.975372	1.931857
H	6.741702	0.289497	2.065280
H	6.922537	1.078423	-0.206663
H	5.050924	1.176545	-1.766258
H	4.586249	2.068831	-0.310488
H	3.867172	-1.645550	-1.628577
H	3.031974	-1.847960	-0.080719
H	7.635029	-2.425459	-0.841139
H	7.894416	-1.511676	0.659558
H	8.302789	-0.795132	-0.913016
H	1.290590	-0.951191	-0.533108
H	-2.301867	3.131656	0.246817
H	-4.410387	2.340530	0.277398
H	-6.698664	1.403640	0.312313
H	-5.127297	-2.534394	-0.227326
H	-2.805446	-1.602968	-0.264356

3e

C	4.616223	-1.557718	0.074465
C	4.645583	-1.094018	1.562245
C	5.176511	0.363162	1.483005
C	5.394034	0.593581	-0.041410

N	5.634709	-0.746813	-0.615587
C	4.133619	1.155694	-0.722119
C	3.250684	-1.260612	-0.581612
C	2.954401	0.224967	-0.550990
C	6.998951	-1.233890	-0.426875
N	1.800818	0.764999	-0.398650
N	0.693015	-0.014267	-0.293498
C	-0.539674	0.573596	-0.159751
S	-0.709283	2.308811	0.051229
C	-2.435713	2.049619	0.139729
C	-2.743057	0.725045	0.003663
N	-1.643048	-0.105719	-0.165448
C	-4.086897	0.123729	0.023844
C	-5.252595	0.896311	0.133467
C	-6.510030	0.313501	0.153017
C	-6.657744	-1.079746	0.059724
C	-5.497142	-1.858517	-0.050715
C	-4.242254	-1.266620	-0.068817
N	-7.918479	-1.672558	0.137871
H	4.843481	-2.619767	-0.038945
H	5.316390	-1.726608	2.147225
H	3.660009	-1.155890	2.028626
H	4.476309	1.086464	1.905255
H	6.116922	0.468552	2.027980
H	6.243539	1.246809	-0.251319
H	4.353282	1.260245	-1.791299
H	3.867894	2.141997	-0.335848

H	3.281821	-1.610096	-1.620566
H	2.474272	-1.830052	-0.060665
H	7.089883	-2.229990	-0.866948
H	7.325555	-1.295596	0.624894
H	7.690959	-0.574541	-0.956123
H	0.692207	-1.002327	-0.516569
H	-3.093157	2.890371	0.285919
H	-5.183556	1.976183	0.200514
H	-7.392386	0.939553	0.243264
H	-5.583352	-2.938724	-0.119409
H	-3.354999	-1.879894	-0.157602
H	-7.985494	-2.593836	-0.269263
H	-8.689557	-1.081819	-0.136820

3f

C	6.011390	-1.893713	0.206241
C	6.184845	-1.261651	1.620291
C	6.916139	0.078715	1.336721
C	7.085029	0.085828	-0.211005
N	7.098579	-1.335178	-0.616204
C	5.886006	0.743706	-0.916318
C	4.670975	-1.477543	-0.437191
C	4.594892	0.027722	-0.590515
C	8.386295	-1.995112	-0.413641
N	3.540419	0.749232	-0.476983
N	2.336698	0.161730	-0.240816
C	1.214886	0.940551	-0.141196

S	1.316989	2.695580	-0.164851
C	-0.418966	2.721666	0.019191
C	-0.925488	1.454151	0.076046
N	0.022798	0.446351	-0.014344
C	-2.342130	1.079913	0.229348
C	-3.372602	2.030050	0.194076
C	-4.699889	1.652632	0.335085
C	-5.038305	0.309603	0.539538
C	-4.022029	-0.649068	0.590227
C	-2.698284	-0.262529	0.421383
N	-6.401828	-0.041495	0.730998
S	-7.184060	-1.302350	-0.074804
O	-8.589133	-1.135441	0.270402
O	-6.465576	-2.537526	0.200282
C	-6.977094	-0.949693	-1.834326
H	6.075722	-2.983571	0.222479
H	6.786789	-1.911577	2.258556
H	5.226625	-1.118029	2.124251
H	6.351460	0.945269	1.686103
H	7.889023	0.107320	1.831647
H	8.007795	0.573734	-0.531440
H	6.062666	0.682853	-1.996704
H	5.785688	1.798336	-0.651254
H	4.597772	-1.951849	-1.423109
H	3.848186	-1.856040	0.177801
H	8.308374	-3.039775	-0.724389
H	8.755811	-1.975611	0.625320

H	9.137037	-1.517115	-1.047266
H	2.184428	-0.836569	-0.320008
H	-0.934600	3.665485	0.083068
H	-3.144378	3.077391	0.035453
H	-5.481936	2.403818	0.286076
H	-4.274629	-1.685516	0.768802
H	-1.911612	-1.004471	0.455138
H	-7.040861	0.728297	0.884439
H	-7.420084	-1.786336	-2.373324
H	-5.912874	-0.877410	-2.054249
H	-7.496279	-0.022411	-2.070657

3g

C	-5.341797	1.728893	0.139691
C	-5.400021	1.212283	1.609069
C	-6.035485	-0.198664	1.477898
C	-6.272079	-0.355045	-0.052968
N	-6.417075	1.019801	-0.575235
C	-5.056912	-0.979943	-0.760682
C	-4.002857	1.359967	-0.535124
C	-3.813684	-0.142981	-0.561499
C	-7.742698	1.596022	-0.362379
N	-2.702102	-0.770364	-0.436134
N	-1.539205	-0.076357	-0.308623
C	-0.356604	-0.758489	-0.198011
S	-0.323826	-2.512455	-0.059243
C	1.412449	-2.402581	0.041570

C	1.821175	-1.100599	-0.037018
N	0.797509	-0.170120	-0.173527
C	3.197945	-0.599977	0.007899
C	4.313219	-1.550640	0.150901
O	5.592303	-1.004034	0.186098
C	5.846865	0.331126	0.096994
C	4.795820	1.251324	-0.039481
C	3.461643	0.733743	-0.080624
C	7.176429	0.743573	0.146187
C	7.463073	2.099115	0.057692
C	6.433030	3.039876	-0.079064
C	5.114972	2.619586	-0.127001
O	4.235561	-2.748140	0.240530
H	-5.492142	2.808010	0.067413
H	-6.021483	1.868996	2.221204
H	-4.411301	1.185071	2.072243
H	-5.389435	-0.986412	1.870110
H	-6.980099	-0.255752	2.022613
H	-7.166356	-0.937623	-0.283330
H	-5.285258	-1.027456	-1.831994
H	-4.861537	-1.996680	-0.413493
H	-4.013279	1.747819	-1.560698
H	-3.186092	1.853860	0.001089
H	-8.480962	1.008560	-0.913194
H	-7.762526	2.611995	-0.764172
H	-8.062010	1.640779	0.692349
H	-1.469766	0.918914	-0.483373

H	2.024084	-3.280864	0.146135
H	2.627006	1.416746	-0.185234
H	7.954281	-0.002021	0.252207
H	8.494665	2.429262	0.095088
H	6.669979	4.094856	-0.147103
H	4.309761	3.338357	-0.232647

3h

C	5.421725	-1.843080	0.085299
C	5.498108	-1.381830	1.572145
C	6.197031	0.002709	1.492318
C	6.448095	0.201151	-0.031501
N	6.532073	-1.159880	-0.601158
C	5.266162	0.905854	-0.720308
C	4.104068	-1.388899	-0.578973
C	3.984778	0.121185	-0.552655
C	7.828584	-1.803598	-0.403752
N	2.902588	0.794272	-0.406610
N	1.709651	0.150728	-0.305164
C	0.556922	0.880157	-0.172493
S	0.596970	2.625724	0.029264
C	-1.146559	2.576327	0.120861
C	-1.610039	1.297233	-0.006637
N	-0.620573	0.338895	-0.172610
C	-3.017676	0.863889	0.018796
C	-4.076697	1.773645	0.155038
C	-5.391636	1.340337	0.178306

C	-5.701517	-0.023304	0.065148
C	-4.656695	-0.944231	-0.073073
C	-3.341470	-0.494256	-0.094547
N	-7.064809	-0.384466	0.097666
C	-7.634754	-1.635287	0.007792
O	-7.004604	-2.667481	-0.119835
C	-9.154851	-1.639054	0.080839
H	5.522350	-2.924784	-0.024655
H	6.087355	-2.087178	2.161675
H	4.510055	-1.326577	2.034124
H	5.584538	0.804682	1.908846
H	7.140354	-0.001356	2.042304
H	7.369551	0.749274	-0.238454
H	5.501246	0.979658	-1.788727
H	5.116859	1.918006	-0.338413
H	4.099848	-1.741156	-1.617367
H	3.263357	-1.862608	-0.062099
H	7.803487	-2.805248	-0.839764
H	8.140328	-1.898752	0.649949
H	8.595936	-1.232591	-0.931758
H	1.593672	-0.832862	-0.517730
H	-1.698551	3.491089	0.259107
H	-3.878452	2.835393	0.242837
H	-6.192072	2.066975	0.284545
H	-4.884208	-1.994891	-0.161486
H	-2.535159	-1.207770	-0.201696
H	-7.704415	0.387650	0.201337

H	-9.457977	-2.270599	0.918236
H	-9.542593	-2.097727	-0.830953
H	-9.604177	-0.650896	0.200126

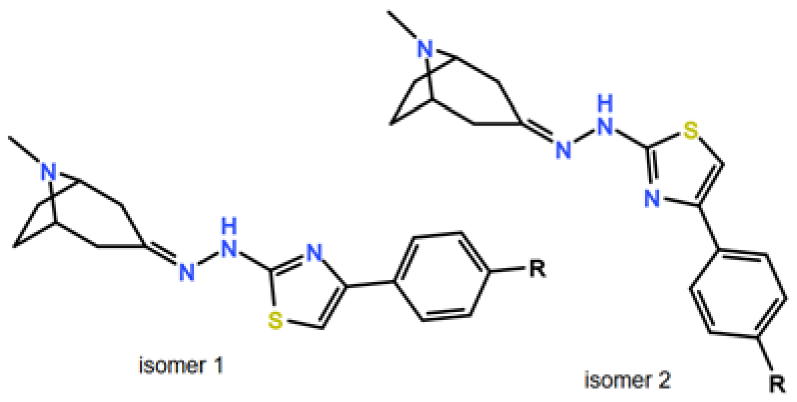

Scheme S1. Structure of isomers 1 and 2 of investigated compounds.

Table S6. Fractional populations of isomers 1 and 2 of investigated compounds [%].

Molecule	isomer 1 [%]	isomer 2 [%]
**3a**	100.00	0.00
**3b**	100.00	0.00
**3c**	100.00	0.00
**3d**	100.00	0.00
**3e**	100.00	0.00
**3f**	99.99	0.01
**3g**	99.99	0.01
**3h**	99.99	0.01
